# Supplementary material for: A nutrient bottleneck controls antibiotic efficacy in structured bacterial populations
Source: Nat Commun. 2026 Feb 20;17:3337. doi: 10.1038/s41467-026-69625-4 (PMC13066622; doi:10.1038/s41467-026-69625-4)
Supplement: Supplementary file 1 — Supplementary Information [file 41467_2026_69625_MOESM1_ESM.pdf]

Supplementary information (SI)

Experimental details

Bacterial strains, growth media, and antibiotics

All experiments are conducted using *E. coli* K-12 substrain MG1655 ΔlacI ΔaraBAD :: $\mathrm{P_{T5}} - \mathrm{gfp} - \mathrm{kanR}$, which constitutively expresses GFP from the chromosome and whose construction has been described previously [1]. Cells are grown in either Luria Bertani (LB) broth or M9 media supplemented with a single carbon source, namely glucose, glycerol, or mannose. LB broth is made by dissolving 2% (w/v) LB powder (Sigma Aldrich) in Milli-Q water for a final concentration of 0.5 g/L sodium chloride (NaCl), 10 g/L tryptone, 5 g/L yeast extract and sterilized by autoclaving. M9 Media is made by dissolving M9 salts (Difco) in MilliQ water and sterilized by autoclaving. After autoclaving, filter-sterilized magnesium sulphate is added such that the final salt concentrations are 6.8 g/L disodium phosphate (anhydrous) (Na_2HPO_4), 3 g/L monopotassium phosphate (KH_2PO_4), 0.5 g/L sodium chloride (NaCl), 1 g/L ammonium chloride (NH_4Cl), and 0.048 g/L magnesium sulphate ($MgSO_4$). Immediately prior to each experiment, filter-sterilized glucose, glycerol, or mannose is added to the reported concentration as a carbon source. Fosfomycin, tetracycline, and colistin stocks are made fresh for each experiment by dissolving fosfomycin-disodium salt, tetracycline hydrochloride, or colistin methanesulfonate (sodium salt), respectively, into autoclaved Milli-Q water and filter-sterilizing. Carbenicillin stocks are made by dissolving carbenicillin disodium salt into autoclaved Milli-Q water and filter-sterilizing then stored at $-20°$C. Carbenicillin stocks are defrosted immediately prior to each experiment. Then, stocks are diluted appropriately into either liquid media or pre-swollen granular hydrogels depending on the experiment.

Preparing the granular hydrogel matrices

We use dense packings of hydrogel grains ("microgels") as growth matrices for bacteria. Each matrix is prepared by dispersing dry granules of internally cross-linked microgels made of biocompatible acrylic acid-alkyl acrylate copolymers (Carbomer 980; Lubrizol, Wickliffe, Ohio) in liquid M9 media (without carbon sources or antibiotics). The granules absorb the liquid until their elasticity prevents further swelling. We ensure a homogeneous dispersion of swollen microgels by mixing for at least 12 h using a rotary mixer, and adjust the final pH to 7.0 by adding 10 M NaOH. Immediately prior to the experiments, glucose, glycerol, mannose, fosfomycin disodium, carbenicillin, tetracycline, or colistin are added to the granular hydrogel at reported concentrations. The swollen microgels are $\sim$5-10 μm in diameter with $\sim$20% polydispersity, but have an internal mesh size of $\sim$40–100 nm, which permits small molecules such as fosfomycin, glucose, and oxygen to freely diffuse throughout while impeding cellular motion [2]. As shown in previous work, the turgor pressure of the growing cells enables them to locally deform the surrounding matrix and thereby lengthen and divide into two daughter cells [3]. In contrast, the stresses exerted by swimming cells are far too small to deform the hydrogel matrix [2, 4], leaving cells trapped within pores equal to or smaller than the cell size. Thus, cells are trapped in the pores of the granular hydrogel matrix to either grow or die in place, but eliminating any effects of chemotaxis or cell motility (Supplementary Fig. 8).

Creating model structured bacterial populations

We construct tunable and well-defined structured bacterial populations by dispersing stationary phase *E. coli* within granular hydrogel matrices and patterning them next to a reservoir of nutrients and antibiotics in the same granular hydrogel matrix. To cleanly isolate just the effects of population structure and 1D nutrient and antibiotic transport, we artificially structure our bacterial populations using granular hydrogel matrices. Since native biofilm-forming strains that control their own matrix production necessarily have feedback on this matrix production (such as at sub-MIC antibiotic levels [5]) and thus community structure that may confound the study of small molecule transport and metabolism alone, we create our model populations with a non-biofilm forming, safe, easily visualizable strain, of MG1655 *E. coli* expressing GFP. The synthetic, biocompatible granular hydrogel matrices physically supporting population structure do not vary spatially or temporally over our experiments, allowing for the precise patterning of populations, and supporting precise control of population cell density across experiments, thus enabling the controlled and reproducible conditions necessary for quantitative comparison with theory.

Additionally, we initialize our population with stationary phase cells, a simplifying choice that does not fully reflect

native biofilms, for three reasons. First, stationary phase cells allow us to create temporal and spatial uniformity in our initial cell population. If we instead used exponential phase cells, we would "shock" the cell population immediately prior to the start of our experiment, likely triggering internal stress responses that may also vary stochastically cell to cell, when resuspending them from a nutrient-rich media supporting exponential phase growth into the carbon-free granular hydrogel. Second, stationary phase cells better allow us to isolate the role of exogenous nutrient supply. To create the cleanest chemical gradients and most controlled experimental system, we wanted the only source of nutrients and antibiotics in our system to be the outer reservoir, which diffuses into a nutrient and antibiotic-free environment containing the cell population. Since exponential phase cells would instead retain intracellular nutrient reserves that could sustain metabolic activity even in nominally nutrient-free conditions, this would confound our ability to precisely control and track nutrient availability through exogenous supply alone. Finally, stationary phase cells are physiologically relevant to many biofilm contexts. While biofilms contain mixed populations, cells in the biofilm interior—where nutrient limitation is most severe—are often in stationary phase or similarly growth-arrested states. Our choice therefore represents a reasonable first approximation for studying the behavior of the nutrient-limited majority of cells in many structured populations, even if it does not capture the full complexity of biofilm heterogeneity.

To make these model structured populations, we grow cells to stationary phase to be resuspended into the swollen granular hydrogel media. Two days prior to each experiment, we pick a single *E. coli* colony from an agar plate and grow it in 2 mL of LB for 24 h in a 37°C shaking incubator. After 24 h, we use 200 μL of this 2 mL culture to inoculate 20 mL of LB, and this larger cell culture is also grown for 24 h in a 37°C shaking incubator, establishing a large volume of stationary phase cells which are used in the experiments. After 24 h of growth, we measure the optical density the stationary phase culture, and use this value to determine the exact volume of cells to spin down for the experiment to reach our desired final cell concentration, since an equivalent optical density of 0.15 corresponds to 10^8 CFU/mL and an equivalent optical density of 1.5 corresponds to 10^9 CFU/mL. We spin the appropriate volume of cells, which ranges from 100 μL - 4 mL depending on the experimental condition, for 15 minutes at 3000 RCF and resuspend the pellet in ∼30 μL of the supernatant. Then, we add this entire cell slurry into an aliquot of the granular hydrogel matrix and evenly disperse the cells by manual shaking to reach the final desired cell concentration. We remove the bubbles introduced by mixing by centrifuging at 3000 RCF for 10 s. This evenly dispersed cell mixture constitutes our model structured cell community. To prepare the nutrient and antibiotic reservoir, we add sterile carbon and antibiotic and stocks into M9 granular hydrogel matrix to their final concentrations for the reservoir. We also add propidium iodide (Sigma Aldrich) to a final concentration of 6 mM in the cell and antibiotic reservoir to act as a dead cell signal.

To pattern the cell community and reservoir, we place a custom fit, 3 mm acrylic divider in the center of each well of a 4 chambered coverglass dish (Cellvis). We gently deposit 0.4 mL of the cell mixture on one side of the divider and 0.4 mL of the nutrient antibiotic reservoir on the other side of the divider with 1 mL syringes with 20 gauge needles attached. Then, we remove the acrylic divider so the two sides collapse together create a smooth interface over which the antibiotics and nutrients can diffuse into the cell population, marking $t = 0$ h for the experiment. Finally, we cover the top surface of the gel with with ∼0.5 mL of parrafin oil to prevent evaporation during imaging.

We confirm the initial cell concentration for each experiment by measuring the colony forming units of the cell mixture within the granular hydrogels. We remove ∼ 100 μL of granular hydrogel and dilute it into 500 mL of PBS in a 1.5 mL tube. We confirm this initial dilution volume by massing the tube before and after adding granular hydrogel. Then, we perform serial 10X dilutions in phosphate buffered saline (PBS) before plating 3, 10 μL droplets of each dilution onto a plate containing 1.5% agar, 2% LB. We incubate the plates at 30°C for ∼16 h before counting colonies and converting to CFU/mL.

Imaging structured bacterial populations and tracking death front position

All time course experiments are imaged every 30-60 minutes using a Nikon A1R+ inverted laser-scanning confocal microscope maintained at 37°C. Multiple images are stitched together to image the entire cell population with a resolution of 0.9 μm/pixel. To image the death front progression, 3 separate z slices are imaged at 50, 100, and 150 μm above the bottom surface of the population and then merged. The death front position is defined as the position at which dead cell propidium iodide signal exceeds a threshold value that is held constant across experimental nutrient and antibiotic conditions for the same cell density and antibiotic. When a death front is not reported, no spatial location ever exceeds the threshold signal value. For imaging heteroresistance, we image at a resolution of 2 μm/pixel and take ∼35 z slice images spaced 100 μm apart to capture the entire 3D population over time. For imaging population clearance by carbenicillin, tetracycline, and colistin populations are imaged on a Nikon A1 confocal microscope with a resolution of 1.2 μm/pixel at a single time point, $t = 20$ h, after incubation in a stationary incubator at 37°C.

Measuring well-mixed growth and death rates to parameterize the model

To understand the joint effects of nutrients and antibiotics on cell growth and death, we perform a series of well-mixed experiments using a Biotek Epoch 2 microplate spectrophotometer. First, cells are grown to stationary phase following the same protocol as for creating the structured bacterial population. Then, we wash the stationary phase cells twice with M9 media by centrifuging for 15 minutes at 3000 RCF, removing the supernatant, and rususpending the pellet into M9 media, to remove any residual nutrients from the overnight growth. After the second wash, we further dilute the washed cells with M9 media to 10X the initial desired concentration for the experiment. We prepare a 96 well plate with serial dilutions of M9 media with defined concentrations of glucose and/or fosfomycin. Then, we inoculate these wells with 15 μL of washed cells for a final well volume of 150 μL. We characterize the bacterial growth and/or death dynamics by incubating the microplate at 37°C with continuous linear shaking at 567 cycles per min over a distance of 3 mm for 16-48 h, measuring UV-vis absorption at 600 nm (OD_{600}) every 10 min. Finally, we verify the starting concentration of the washed cell inoculum by serially diluting and plating cells on 2% LB 1.5% agar before incubating overnight and counting colonies, similar to the protocol for experiments on structured bacterial populations. For well-mixed experiments measuring the minimum inhibitory concentrations of carbenicillin, tetracycline, and colistin, we follow the same procedure but measure optical density over time using a Tecan Spark spectrophotometer.

Testing for fosfomycin degradation in the presence and absence of cells

To test if there is any loss of potency for fosfomycin from incubation at 37°C in M9 media without cells, we repeat bacterial growth and death measurements in fosfomycin that was pre-incubated compared to fresh stock (Supplementary Fig. 9A). Both 48 and 24 hours prior to the experiment, we prepare a stock solution of fosfomycin, filter-sterilize it, and dilute it to our working concentration in M9 media supplemented with 24.4 mM glucose (which would dilute to 22.2 mM glucose after cell stocks are added). We incubate the fosfomycin-glucose M9 media at 37°C in a stationary incubator for 48 and 24 h respectively. Then, we prepare serial dilutions with the pre-incubated stocks as well as freshly prepared fosfomycin in a 96-well microplate. We inoculate these cultures with washed stationary phased cells of two different densities which are prepared as previously described for other microplate measurements. Following inoculation, we incubate the microplate at 37°C with continuous linear shaking at 567 cycles per min over a distance of 3 mm for 16 h, measuring UV-vis absorption at 600 nm (OD_{600}) every 10 min with a Biotek Epoch 2 plate reader. We compare the growth and death dynamics for cells in freshly made or old fosfoymcin and saw no difference, indicating no degradation or loss of potency over the time scales of our experiment in the absence of cells.

To additionally test if there is any loss of potency for antibiotic from incubation at 37°C in M9 media in the presence of cells, we repeat the above experiment for fosfomycin that was pre-incubated with a dense, growing cell population (Supplementary Fig. 9B). Twenty-four hours prior to the experiment, we prepare a stock solution of fosfomycin, filter-sterilize it, and dilute it to 2X our working concentration in M9 media supplemented with 22.2 mM glucose either with or without a 10^8 CFU/mL population of stationary phase cells. To prepare the stationary phase population of *E. coli* we follow the protocol outlined above for cell preparation in the model structured population, but here we add our washed cells into liquid M9 media supplemented with 22.2 mM glucose instead of of granular hydrogel. Twenty-four hours prior to the experiment, we also prepare an additional culture of 10^8 CFU/mL population in in M9 media supplemented with 22.2 mM glucose without glucose, to act as a control volume of pre-digested media. Then, we incubate both cultures at 37°C with a shaking incubator for 24 hours. The day of the experiment, we spin down the cells from antibiotic and filter sterilize the pre-digested media through a 0.22 μm filter. Then, we prepare a microplate with equal volumes of antibiotic-free pre-digested media and fresh M9 media with 22.2 mM glucose. We serially dilute either freshly made fosfomycin, fosfomycin incubated wihtout cells, or fosfomycin incubated with cells. We inoculate these cultures with washed stationary phased cells of one initial density which are prepared as previously described for other microplate measurements. Following inoculation, we incubate the microplate at 37°C with continuous linear shaking at 567 cycles per min over a distance of 3 mm for 16 h, measuring UV-vis absorption at 600 nm (OD_{600}) every 10 min with a Biotek Synergy H1 plate reader. We found no difference in fosfomycin efficacy governing growth and death dynamics across these three conditions, supporting our existing model assumption that there is not substantial loss or degradation of fosfomycin over the time scales of our experiments, even in the presence of growing cell populations.

Testing small molecule diffusion

To confirm that there are no other transport mechanisms besides diffusion for small molecules in the granular hydrogel matrix, we measure the diffusive spreading of a representative fluorescent small molecule, fluorescein, over time. We pattern our granular hydrogel matrices, which are swollen in the same M9 liquid media for cell based experiments, to mimic diffusion of a solute between two semi-infinite planar domains. That is, on one half of the dish we had a source of fluorescein at an initial concentration of $c_1 = 50$ µM. At $t = 0$ h, this source concentration diffuses into a reservoir initially at $c_2 = 0$ µM. We image the fluorescent signal every 10 minutes for 16 hours with a resolution of 2.16 µm/px and take image intensity to be a proxy for local fluorescein concentration, thereby normalizing the data so our maximum signal is $c_1 = 50$ µM. To confirm a fully diffusive profile, we successfully collapse the data across all $\frac{x-x_0}{\sqrt{t}}$. Additionally, we fit the data from three independent replicates to the analytical solution for diffusion between two semi-infinite planar domains, $c(x,t) = (\frac{c_1+c_2}{2}) - (\frac{c_1+c_2}{2})\mathrm{erf}(\frac{x}{2D\sqrt{t}})$ where $\mathrm{erf}(x) = \frac{2}{\pi}\int_0^x -y^2\,dy$ to obtain an estimate of the diffusion coefficient, $D = 2.2 \pm 0.1$ mm^2/h. This value is in good agreement with literature values for fluorescein diffusion in water [6–8], as we expect in our granular hydrogel system with large interstitial pore space.

Quantifying heteroresistance

The heteroresistance profile of the starting cell population, or the frequency of resistant subpopulations able to grow at varying antibiotic concentrations, was quantified using standard methods of population analysis profiling [9, 10]. Briefly, cells are grown to stationary phase following the same protocol as for creating the structured bacterial population or measuring well mixed growth and washed once with M9 media. Then, we perform 10X serial dilutions of the washed cells and plate 3, 10 µL droplets each 10X dilution onto plates containing 1.5% agar, M9 media with 22 mM glucose, and 2 fold increments of fosfomycin ranging from 0.5-512 µg/mL. The number of resistant mutants is compared to cells from the same culture grown on antibiotic-free plates to give the frequency of resistant bacterial subpopulations. Resistant subpopulations with frequencies greater than 10^{-7} were detected over an 8-fold concentration range above the highest non-inhibitory concentration of fosfomycin, marking initial cell population of our experiments heteroresistant to fosfomycin.

Developing, parameterizing, and simulating the model

We use the experiments described below to formulate and parameterize the following 1D continuum model of our experiments.

$$\text{Bacteria}: \frac{\partial b}{\partial t} = \begin{cases} \overbrace{bg\frac{c}{c+c_*}}^{\text{Growth}}, & b < \Phi b_0 \\ \underbrace{b\Big(\frac{c}{c+c_*}\Big)\Big(\frac{(g+d)a_*}{a+a_*} - d\Big)}_{\text{Growth\&Death}}, & b > \Phi b_0 \end{cases} \tag{1}$$

$$\text{Nutrient}: \frac{\partial c}{\partial t} = \underbrace{D_c\nabla^2 c}_{\text{Diffusion}} - \underbrace{b\kappa\frac{c}{c+c_*}}_{\text{Consumption}} \tag{2}$$

$$\text{Antibiotic}: \frac{\partial a}{\partial t} = \underbrace{D_a\nabla^2 a}_{\text{Diffusion}} \tag{3}$$

Since the interstitial mesh of our granular hydrogel (∼100 nm) is much larger than both glucose and fosfomycin (∼1 nm), we approximate the diffusivity of each of these small molecules to be uniform and equivalent to the diffusivity of glucose in water $D_c = D_a$ [11], as has been done previously for granular hydrogel systems [3, 12, 13]. For the antibiotic, there is no substantial degradation or loss of potency over the timescale of our experiments in the presence or absence of cells (Fig. S5), and the effects of cell uptake are negligible (see calculation below), so the dynamics of the antibiotic are entirely captured by diffusion (Eq. 3). In contrast, nutrients are depleted by cells in the system.

The growth of bacteria on single carbon sources is well understood by Monod kinetics [14–16], and this growth and consumption interaction forms the basis for our model, as detailed further below.

Maximum growth rate

We measure the maximum exponential growth rate of *E. coli* grown in M9 media with 22 mM glucose, as shown in Supplementary Fig. 10. We fit an exponential curve to the region over hours 4-9 for 8 replicates across four independent experiments to obtain an average maximum growth rate of 0.32 h^{-1}.

Nutrient consumption rate per cell

Next, we fit the nutrient consumption rate per cell using yield measurements. We grow cells cultures with varying initial cell density in a range of glucose concentrations, following the protocol for plate reader growth. Then, after 20 h of growth in a shaking plate reader, we measure the cell density (CFU/mL) of each sample, as shown in Supplementary Fig. 11A. To estimate the nutrient consumption rate per cell from these measurements, we again turn to the well established Monod model for bacterial growth on single, consumable carbon sources. Bacteria $b(t)$ grow from an initial density b_0 according to the equation $\frac{db}{dt} = bg\frac{c}{c+c_*}$, where g is the maximum growth rate and $c(t)$ is the local nutrient concentration. Nutrients $c(t)$ are simultaneously depleted from their initial concentration c_0 according to equation $\frac{dc}{dt} = -\kappa b\frac{c}{c+c_*}$, where κ is the maximum consumption rate per cell. For simplicity, we assume that cells are always growing and consuming nutrients at the maximal rates g and κ, thus setting the $\frac{c}{c+c_*}$ Monod term to 1. Thus, bacterial density over time obeys the equation $b = b_0 e^{gt}$. Cell reach a final density of b_f in time $t_* = \frac{1}{g}\ln(\frac{b_f}{b_0})$. Assuming that cells reach this final density once they have completely exhausted nutrients from initial value of c_0 to 0, we can integrate the equation for nutrients $\frac{dc}{dt} = -\kappa b_0 e^{gt}$ from c_0 to 0 in the time 0 to t_*. By solving for κ, we obtain that $\kappa = \frac{c_0 g}{b_f}$. Calculating κ from these measurements in Supplementary Fig. 11A gives a value of $\kappa = 1.1 \times 10^{-9} \pm 1.3 \times 10^{-10}$ (mM)(CFU/mL)$^{-1}$(h)$^{-1}$.

To confirm that this value matches the dynamics we see in plate reader growth, we compare simulated well mixed growth to independent experiments measuring cell growth over time in a range of glucose concentrations. First, we convert the OD_{600} values from the plate reader to CFU/mL using a linear conversion rate (Supplementary Fig. 11B). Then, we simulate well-mixed growth according to unapproximated Monod kinetics across 4 nutrient conditions (Eqn. 1-3 without diffusion terms) and compare these results to experimental measurements seeing good agreement with our cell yield estimation.

In the model, we assume that the consumption rate of nutrients is not impacted by local antibiotic concentration, based on prior work [17]. For simplicity, we also omit any lag time and assume that cells begin growing and consuming nutrients according to Monod kinetics immediately. We justify this simplification of lag time omission with three lines of evidence. First, there is timescale separation. Stationary phase E. coli typically resume growth within 1 h upon nutrient addition, short compared to death front progression timescales (6-24 hours). Second, we observe quantitative agreement. Our model reproduces experimental observations across multiple conditions without including lag time. If lag time were dominant, we would expect systematic early-time discrepancies. The close agreement suggests lag time is either negligible or implicitly incorporated into our measured growth rates (also parameterized using stationary phase cells). Third, to reduce complexity cost. Explicitly including lag time would require spatially- and temporally-dependent parameters, adding significant complexity without improving model-experiment agreement.

Growth threshold and piecewise $\partial b/\partial t$ formulation

To understand how the presence of fosfomycin influences cell growth and death rates across nutrient conditions, we culture bacteria in a range of glucose and fosfomycin concentrations and measure cell growth and death via UV vis spectroscopy. In high nutrient environments, as shown in Main Text Fig. 3B, we observe that at first cells in both antibiotic rich and poor environments grow exponentially ($t = 0 - 2$ h). Only after this initial period of growth to a threshold cell density relative to the initial cell density, $b > \Phi b_0$, do cells begin to die exponentially in high antibiotic environments ($a > a_*$), similar to what has been previously reported for β-lactam antibiotics which also target cell wall biosynthesis [18]. Prior work by Kim et al. [18] showed that as an *E. coli* cell lengthens in preparation for division, more errors in newly synthesized cell wall are introduced and the probability of lysis increases. Thus, cells must lengthen and biomass must accumulate relative to the starting population density (equivalent to a value of $b > b_0$ at the time of lysis) to incorporate those defects in the cell wall that increase the probability of lysis. We

hypothesize that similar behavior might be at play for our system with fosfomycin, which targets another step of cell wall biosynthesis: stationary phase *E. coli* cells need to grow to an average threshold cell density relative to the initial cell density, $b > \Phi b_0$, before local antibiotic causes cell death and lysis. If that threshold density is never reached, as is the case for our nutrient-poor conditions that support little biomass increase (Main Text Fig. 3C), we hypothesize that there has not been sufficient damage to newly synthesized cell wall by the antibiotic to cause lysis, so cells remain alive once nutrients are depleted. Therefore, mathematically, we chose to describe this transition in behavior from exponential growth to exponential death in high nutrient, high antibiotic environments in the simplest way possible: with a piecewise function for $\frac{\partial b}{\partial t}$ that transitions at a given value of $\Phi = b/b_0$. So, the first rule for bacteria in our model is that, initially, cells grow exponentially according to only Monod kinetics until reaching the threshold density, Φb_0 (Eq. 1a). The growth threshold parameter Φ thus describes the threshold cell density (relative to the initial cell density) that cells must grow to in order to be sensitized to the effects of local antibiotic and transition from an initial period of exponential growth to a period of exponential death. We use this piecewise model for $\frac{\partial b}{\partial t}$ which transitions at $b > \Phi b_0$ in our spatial model because we observe two different behaviors, either exponential growth (positive $\frac{db}{dt}$) or exponential death (negative $\frac{db}{dt}$), for the same local nutrient and antibiotic concentrations depending on the timing of exposure in simplified, well-mixed conditions. Indeed, for spatially extended, immobilized populations beginning from a spatially uniform initial condition, the ratio $\Phi = b/b_0$ effectively tracks this local growth history at each position. We also note that the observed sharpness in the death front in our spatial simulations, which matches our experimental findings (Main Text Fig. 1D $t = 8 - 24$ h), does not arise solely from the piecewise structure of our model. Rather, it emerges from the coupling between local nutrient depletion by growing cells, the resulting spatial lag between nutrient and antibiotic penetration (Main Text Fig. 3G, panel iii), and the growth threshold requirement before death can occur detailed further below. Therefore, other biological parameters such as the death rate, population cell density, and applied nutrient concentration and diffusivity also influence the sharpness of the death front in our model.

To determine the value of Φ, we culture cells in a range of nutrient concentrations for 0 and 256 µg/mL Fos-Na. We compare initial cell density to final cell density and identify the critical initial nutrient level c_{crit} below which no cell death is observed for each initial cell density (dashed lines in Supplementary Fig. 12A, y-axis values of dots in Supplementary Fig. 12B). As expected, the critical nutrient level is linearly dependent on initial cell concentration b_0 (Supplementary Fig. 12B) and we can use this linear relation between b_0 and c_{crit} to parameterize Φ according to the following calculations.

According to the well-established model of Monod kinetics used above to calculate nutrient consumption rate per cell, we can calculate the time t_{crit} that it takes for bacteria to grow from an an initial density b_0 to a final density Φb_0. Assuming that bacteria always grow at the maximal exponential rate g, $t_{crit} = \frac{\log\Phi}{g}$. During this period of growth, bacteria are likewise depleting nutrients according to the equation $\frac{dc}{dt} = -\kappa b_0 e^{gt}$. The minimum initial nutrient concentration c_{crit} that supports the growth of the population from b_0 to Φb_0 is the amount of nutrients depleted by the growing population in time t_{crit}. Thus, we can integrate this equation from time $t = 0$ to $t = t_{\text{crit}}$ where nutrients drop from an initial concentration of $c = c_{\text{crit}}$ to $c = 0$ and solve for c_{crit} to obtain the expression $c_{crit} = \frac{\kappa b_0}{g} e^{g t_{\text{crit}}}$. By substituting for $t_{crit} = \frac{\log\Phi}{g}$, we obtain the linear expression that $c_{\text{crit}} = \frac{\Phi \kappa b_0}{g}$. Computing Φ for each experiment (1 dot in Supplementary Fig. 12B represents 1 experiment shown in Supplementary Fig. 12A), gives $\Phi = 1.5 \pm 0.3$.

Finally, we simulate well-mixed growth (Eq. 1-3 from the main text, omitting diffusion) using the measured value of $\Phi = 1.5$ for two different cell densities and compare these simulation results to experimental time-course measurements, seeing good qualitative agreement (Supplementary Fig. 12C). In the simulations, cells reach the threshold density sooner than in experiments, which is expected because our simulations do not include a lag time during which cells grow below their maximum exponential growth rate even if $c > c_*$.

Critical antibiotic concentration and maximum death rate

To understand the joint effects of local nutrient and antibiotic concentration on cell growth and death, we culture cells in a range of nutrient and antibiotic concentrations and measure optical density with UV/Vis spectroscopy. In all conditions where initial glucose c_0 is larger than c_{crit}, we first observe a uniform biomass increase no matter the local antibiotic concentration (Main Text Fig. 3A and Supplementary Fig. 12C). After this initial period of growth, cells either continue growing exponentially at a maximal growth rate g for low antibiotic concentrations or begin dying exponentially at a maximal death rate d for high antibiotic concentrations. We define the net exponential growth or death rate as γ where $\frac{db}{dt} = b\gamma$. In the limit of no antibiotics, growth is only dependent on nutrient concentration, so $\gamma = g\frac{c}{c+c_*}$, or traditional Monod kinetics.

To measure $\gamma = f(c, a)$ across experimental conditions, we fit an exponential curve over sliding 2 hour time windows between 1.5 and 9 h of growth for each experimental replicate in each nutrient-antibiotic condition. If any of the measured rates over the sliding time window are less than -0.1h^{-1}, we consider that cells die exponentially and

take the lowest measured rate over the time window as the reported net growth and death rate γ. Next, if biomass does not change, or all measured rates fall between $-0.1 - 0.1\text{h}^{-1}$, we take the average rate over the time window to be γ. We choose these thresholds to account for the noise that occurs when measuring optical density of dilute bacterial suspensions. Finally, if there is a positive biomass increase over this time window, we report the maximum rate as γ for that condition. All measured net growth and death rates γ across conditions can be seen in the dots in Supplementary Fig. 13, which represent 192 replicates across over 96 glucose and fosofmycin conditions for each cell density.

Upon inspection of the data, we first observe that below a threshold nutrient concentration no growth or death is observed (Supplementary Fig. 13, white points below horizontal dashed line). Above this threshold nutrient concentration, we measure a positive γ (growth) at low antibiotic concentrations (Supplementary Fig. 13, green points) and a negative γ (death) at high antibiotic concentrations (Supplementary Fig. 13, purple points). The threshold nutrient concentration corresponds to $c_0 = c_{\text{crit}}$ that supports growth of b_0 to Φb_0 for each cell density we tested (Supplementary Figs. 13A vs. 13B). The transition between exponential growth and death occurs at a consistent critical antibiotic concentration a_* across nutrient levels (Supplementary Fig. 13, vertical dashed line). To describe this transition from exponential growth to death in the limit of high nutrient levels ($c >> c_*$, $\frac{c}{c+c_*} = 1$), we use an inverse Monod function for antibiotic namely that $\gamma = f(a) = \frac{(g+d)a_*}{a+a_*} - d$. Combining the nutrient and antibiotic dependence for γ by multiplying the two Monod terms yields $\gamma = \left(\frac{c}{c+c_*}\right)\left(\frac{(g+d)a_*}{a+a_*} - d\right)$ and $\frac{db}{dt} = b\gamma = b\left(\frac{c}{c+c_*}\right)\left(\frac{(g+d)a_*}{a+a_*} - d\right)$ which effectively describes the net bacterial growth or death rate in terms of local nutrient and antibiotic concentration. The multiplication of these two Monod functions follows prior literature describing the joint effects of nutrient and antibiotic [19] and incorporates the broader microbiological phenomenon that maximum death rate varies linearly with maximum growth rate [20].

To parameterize a_* and d from these data, we fit the data for each cell density to the term $\gamma = \left(\frac{c}{c+c_*}\right)\left(\frac{(g+d)a_*}{a+a_*} - d\right)$ (Supplementary Fig. 13, background color). We fix the maximum growth rate $g = 0.32\ \text{h}^{-1}$ and obtain fit values for a_*, d, and c_*. For the simulations, we use parameters from the low cell density data set because they more accurately measure faster maximum growth and death rates at extreme conditions whereas the high cell density data is bounded by the saturating values in optical density measurements. Notably, however, a_* does not change appreciably across cell density. Additionally, since the acquired data actually fits c_{crit} not c_* for the critical nutrient value (increases $\sim$10 fold for a 10 fold cell density change), we use the well established literature Monod value for *E. coli* growth on glucose $c_* = 0.02$ mM in our simulations [14]. Indeed, UV-vis measurements are not sensitive enough to detect a biomass increase for initial cell populations dilute enough to appreciably grow on $c_0 = c_*$. A summary of all parameter values that match our experimental conditions can be found in Supplementary Table 1.

Estimating antibiotic loss into cells

We estimate the cell concentration dependent loss of fosfomycin from the bulk as follows. First, prior work demonstrates that intracellular fosfomycin concentration equilibrates to the extracellular concentration within minutes of antibiotic exposure [21]. Thus, the fraction of intracellular fosfomycin compared to the bulk concentration can be approximated by the volume fraction occupied by cells. A single *E. coli* cell has a volume of approximately 1 μm^3, so a dense cell population of 10^9 CFU/mL, 10X denser than the cell concentrations tested experimentally, occupies a total volume fraction of just 0.1%. Thus, only 0.1% of applied fosfomycin is lost into cell cytoplasm by this estimation. Additionally, if we consider that fosfomycin binds irreversibly to the intracellular target protein, MurA [22], we can approximate the molecules of fosfoymin lost into cells as the total concentration of MurA proteins. In exponential phase, cells express the maximum concentration of $\sim$ 1000 MurA/cell [23]. So, a 10^9 CFU/mL cell population has $\sim 10^{12}$ molecules MurA/mL which equates to 0.0002 μg/mL bound fosfomycin compared to bulk concentrations of 16-2000 μg/mL fosfomycin in our experiments. By both of these metrics, the concentration of fosfomycin lost into cells, even at high cell densities, is negligible. Thus, we omit cell uptake terms from our model and entirely capture the dynamics of fosfomycin by diffusion.

Numerically simulating the model

To simulate the continuum reaction-diffusion equations of our system, we discretize our system and implement a forward-time, centered-space scheme of finite differences. For all simulations, the temporal and spatial resolution of the simulations is 1×10^{-4} h and 0.025 mm, respectively. Repeating representative simulations with varying spatial and temporal resolution reveals that finer discretization does not appreciably alter the results (Supplementary

Fig. 16). Thus, our choice of discretization is sufficiently resolved such that the results are not appreciably influenced by discretization.

For numerical simulations exactly recapitulating the experiments (Main Text Fig. 3E-G), we set the total simulation domain size L to 19.9 mm, matching the dimension of the experimental dish and run simulations for 24 h, matching the duration of imaging. For simulations sweeping across biophysical conditions (Main Text Fig. 4A-D), we use a larger system size L of 80 mm, ensuring that $L/2$, the size of the bacterial population and maximum position of the death front, is always larger than the simulated death front position at the final time points so that edge effects are minimal. Further, when evaluating front position 12 h after forming, we vary the total simulation time to be either 24 h or at least 20 h after a predicted death front would appear (see below for calculations of the predicted time for a front to appear) for cases with slower growth and death rates. Finally, to match the closed nature of our experimental system, we impose no flux boundary conditions on each edge of the simulated dish.

When sweeping over many biophysical conditions, we randomly sample parameter values across the possible range shown in Supplementary Table 2, while applying the following constraints. First we restrict that cells must die faster than they grow ($d < g$) to prevent biomass accumulation. Next, c_0/c_* and a_0/a_* must greater than or equal to 5 to ensure possible theoretical front formation. While non-linearity in the Monod function allows for modest death front formation at lower c_0/c_* and a_0/a_* values, the theoretical maximum front prediction breaks down. Finally, for data shown in Main Text Fig. 4D, $D_c = D_a$ and $c_0/c_* = a_0/a_*$ such that the theoretical maximum front position is neither antibiotic nor nutrient limited, rather falling along the white diagonal line of Fig. 4A. When freely simulating all parameters without these constraints, the data still collapse according to our predicted $\mathcal{S}$ parameter, though less tightly (Supplementary Fig. 14). In all cases, we define the death front position to be the deepest spatial location where $b(x) < \frac{1}{2}b_0$ at each time point. All numerical simulations were performed using MATLAB R2024a.

Theoretical calculation of death front dynamics

To predict how far a death front could theoretically penetrate into a structured bacterial population, we considered how far both nutrients and antibiotics can diffuse from the reservoir into the cell population in a given amount of time. The concentration profile for a solute diffusing between 2 semi-infinite planar domains with concentrations c_1 and c_2 ($c_1 > c_2$) can be described by the following function: $c(x,t) = (\frac{c_1+c_2}{2}) - (\frac{c_1+c_2}{2})\text{erf}(\frac{x}{2D\sqrt{t}})$ where $\text{erf}(x) = \frac{2}{\pi}\int_0^x -y^2\,dy$. For the experimental system geometry, $c_1 = c_0$ and $c_2 = 0$. Thus the diffusive profile of nutrients and antibiotics over the system geometry $-L/2 < x < L/2$ can be described as: $c(x,t) = (\frac{c_0}{2}) - (\frac{c_0}{2})\text{erf}(\frac{x}{2\sqrt{D_c t}})$.

Without nutrient consumption or other loss, cells will start the growth sensitization and death process at a given position once both nutrients and antibiotics have reached that position locally. Since both cell growth and death obey Monod functions, which sigmoidally step up from a value of 0 to 1 at the critical concentration c_*, we approximate that growth sensitization and death occur once the local concentration reaches c_*. A death front is detected at a given position once cells at that position have grown exponentially from an initial concentration b_0 to the growth threshold ϕb_0 and then died exponentially from ϕb_0 to a local cell density of $\frac{1}{2}b_0$, at which point a death front is detected. The total time delay for growth and death $t_{\text{grow+die}}$ can be approximated as the characteristic time for growth t_g plus the characteristic time for death t_d. For simplicity, we assume that cells always grow and die at their maximum exponential rates. Then, exponentially growing cells obey the function $\frac{db}{dt} = gb$. To find t_g, we integrate each side of the equation $\int_{b_0}^{\Phi b_0} \frac{1}{b}\,db = \int_0^{t_g} g\,dt$ and solve for $t_g = \frac{\ln(\Phi)}{g}$. Similarly, exponentially dying cells obey the function $\frac{db}{dt} = -db$. To find t_d, we integrate each side of the equation $\int_{\Phi b_0}^{\frac{1}{2}b_0} \frac{1}{b}\,db = \int_0^{t_d} -d\,dt$ and solve for $t_d = \frac{\ln(2\Phi)}{d}$. Thus, $t_{\text{grow+die}} = \frac{\ln(\Phi)}{g} + \frac{\ln(2\Phi)}{d}$. Then to find the maximum death front position after a given time T, we solve for the position x_{front} where $c(x_{\text{front}}, t_{\text{eval}}) = c_*$ where $t_{\text{eval}} = T - t_{\text{grow+die}}$ to account for the time delay of growth and death. Since both nutrient and antibiotic must reach their local threshold concentrations for the death front to reach a given position, we take the minimum diffusion distance between the two solutes as the maximum possible death front x_{front} position after a given time T.

$$x_{\text{front}} = \min \begin{cases} 2\sqrt{D_c t_{\text{eval}}}\,\text{erf}^{-1}(1 - \frac{2c_*}{c_0}) \\ 2\sqrt{D_a t_{\text{eval}}}\,\text{erf}^{-1}(1 - \frac{2a_*}{a_0}) \end{cases}$$

As shown in Main Text Fig. 4A, we see good agreement between this theoretical prediction and numerical simulations of our model. The nonlinearity of the Monod function in the simulation means a front can form and progress even if $a_0 < 2a_*$ or $c_0 < 2c_*$, and thus the theoretical predictions slightly deviate for low $\frac{c_0}{c_*}$ and $\frac{a_0}{a_*}$.

Deriving the dimensionless slowdown parameter $\mathcal{S}$

To understand exactly how and when nutrient consumption impacts death front propagation, we consider two characteristic time scales: the time for the initial cell population to deplete source nutrients t_{consume} and the time scale of growth sensitization and death $t_{\text{grow+die}}$. We compare these two timescales to obtain a dimensionless death slowdown parameter $\mathcal{S} = \frac{t_{\text{grow+die}}}{t_{\text{consume}}}$.

We previously calculate the term $t_{\text{grow+die}} = \frac{\ln(\Phi)}{g} + \frac{\ln(2\Phi)}{d}$ in the section above. Now, to calculate t_{consume}, we consider the time it takes for a fixed cell population to deplete local nutrients. Bacteria $b(t)$ grow from an an initial density b_0 according to the equation $\frac{db}{dt} = bg\frac{c}{c+c_*}$. Nutrients $c(t)$ are simultaneously depleted from their initial concentration c_0 according to equation $\frac{dc}{dt} = -\kappa b\frac{c}{c+c_*}$. For simplicity, we assume that the cell population size is fixed at $b = b_0$ and that these cells are always are always consuming nutrients at the maximal rates κ, thus setting the $\frac{c}{c+c_*}$ Monod function to 1. So, the nutrient concentration over time obeys the equation $\frac{dc}{dt} = -\kappa b_0$. Next, we integrate each side to find the time t_{consume} that it takes to deplete nutrients from an initial concentration c_0 to 0: $\int_{c_0}^{0} db = \int_0^{t_{\text{consume}}} -\kappa b_0 \, dt$. Solving for t_{consume} gives $t_{\text{consume}} = \frac{c_0}{\kappa b_0}$.

The dimensionless slowdown parameter $\mathcal{S}$ compares these two timescales such that $\mathcal{S} = \frac{t_{\text{grow+die}}}{t_{\text{consume}}} = \frac{\left(\frac{\ln(\Phi)}{g} + \frac{\ln(2\Phi)}{d}\right)\kappa b_0}{c_0}$. When $\mathcal{S}$ is small, cells grow and die must faster than they consume and deplete nutrients so the death front can progress at its maximal diffusive pace. When $\mathcal{S}$ is large, nutrients are consumed and depleted faster than cells can grow and die. Thus, cells are locally starved and can remain tolerant despite high local antibiotic concentration, effectively slowing down bacterial clearance by the antibiotic—the nutrient bottleneck effect.

Supplementary movies

Supplementary movies are available on Zenodo (`https://zenodo.org/records/14990206`).

Movie 1. Time-lapse microscopy of a 10^8 CFU/mL cell population being treated with 2048 μg/mL Fos-Na and 0 mM glucose. Green GFP signal corresponds to live cells with intact cell membranes. Magenta signal is propidium iodide, a dead cell indicator. Three biological replicates are shown and scale bar is 1 mm.

Movie 2. Time-lapse microscopy of a 10^8 CFU/mL cell population being treated with 2048 μg/mL Fos-Na and 0.22 mM glucose. Green GFP signal corresponds to live cells with intact cell membranes. Magenta signal is propidium iodide, a dead cell indicator. Three biological replicates are shown and scale bar is 1 mm.

Movie 3. Time-lapse microscopy of a 10^8 CFU/mL cell population being treated with 256 μg/mL Fos-Na and 0.22 mM glucose. Green GFP signal corresponds to live cells with intact cell membranes. Magenta signal is propidium iodide, a dead cell indicator. Three biological replicates are shown and scale bar is 1 mm.

Movie 4. Time-lapse microscopy of a 10^8 CFU/mL cell population being treated with 2048 μg/mL Fos-Na and 2.2 mM glucose. Green GFP signal corresponds to live cells with intact cell membranes. Magenta signal is propidium iodide, a dead cell indicator. Three biological replicates are shown and scale bar is 1 mm.

Movie 5. Time-lapse microscopy of a 10^9 CFU/mL cell population being treated with 2048 μg/mL Fos-Na and 0.22 mM glucose. Green GFP signal corresponds to live cells with intact cell membranes. Magenta signal is propidium iodide, a dead cell indicator. Three biological replicates are shown and scale bar is 1 mm.

Movie 6. Numerical simulation of a 10^8 CFU/mL cell population being treated with 2048 μg/mL Fos-Na and 0.22 mM glucose. Y axis is normalized by initial concentration of cells, nutrient, and antibiotic.

Movie 7. Time-lapse microscopy of a 10^8 CFU/mL cell population being treated with 64 μg/mL Fos-Na and 2.2 mM glucose. Green GFP signal corresponds to live cells with intact cell membranes. Magenta signal is propidium iodide, a dead cell indicator. Three biological replicates are shown and scale bar is 1 mm.

Movie 8. Time-lapse microscopy of a 10^8 CFU/mL cell population being treated with 16 μg/mL Fos-Na and 2.2 mM glucose. Green GFP signal corresponds to live cells with intact cell membranes. Magenta signal is propidium iodide, a dead cell indicator. Three biological replicates are shown and scale bar is 1 mm.

Movie 9. Time-lapse microscopy of a 10^9 CFU/mL cell population being treated with 64 μg/mL Fos-Na and 2.2 mM glucose. Green GFP signal corresponds to live cells with intact cell membranes. Magenta signal is propidium iodide, a dead cell indicator. Three biological replicates are shown and scale bar is 1 mm.

TABLE S1: Values of model parameters used to match experiments.

Parameter	Units	Values	Reference (or) Rationale for choice
Maximal growth rate, g	h^{-1}	0.32	This study
Maximal death rate, d	h^{-1}	0.71	This study
Nutrient consumption rate per cell κ	$(mM)(CFU/mL)^{-1}(h)^{-1}$	1.1×10^{-9}	This study
Critical nutrient concentration, c_*	mM	0.02	[14]
Critical antibiotic concentration, a_*	μg mL^{-1}	7	This study
Nutrient diffusivity, D_c	mm^2 h^{-1}	2.2	[11]
Antibiotic diffusivity, D_a	mm^2 h^{-1}	2.2	$D_a = D_c$ since both are similarly sized small molecules
Growth Threshold, Φ	−	1.5	This study
Initial bacteria concentration, b_0	CFU mL^{-1}	$10^8 - 10^9$	This study
Initial glucose concentration, c_0	mM	$0.22 - 2.2$	This study
Initial fosfomycin concentration, a_0	μg mL^{-1}	$256 - 2048$	This study

TABLE S2: Ranges of values of model parameters explored in this study with corresponding references.

Parameter	Units	Range	References (or) Rationale for choice
Maximal growth rate, g	h^{-1}	$0.06 - 2$	[24–27]
Maximal death rate, d	h^{-1}	$0.1 - 4$	Death rate scales linearly with growth rate [26]
Nutrient consumption rate per cell κ	$(mM)(CFU/mL)^{-1}(h)^{-1}$	$1 \times 10^{-9} - 5 \times 10^{-8}$	[3, 28]
Critical nutrient concentration, c_*	mM	$2 \times 10^{-4} - 0.5$	[14, 27]
Critical antibiotic concentration, a_*	μg mL^{-1}	$0.1 - 512$	Approximated by MIC [29]
Nutrient diffusivity, D_c	mm^2 h^{-1}	$0.3 - 3$	[30–32]
Antibiotic diffusivity, D_a	mm^2 h^{-1}	$0.01 - 2$	[33, 34]
Growth Threshold, Φ	−	1.5	This study
Initial bacteria concentration, b_0	CFU mL^{-1}	$4 \times 10^4 - 2 \times 10^{11}$	[3, 35–37]
Initial glucose concentration, c_0	mM	$0 - 48$	[38–40]
Initial fosfomycin concentration, a_0	μg mL^{-1}	$0 - 1500$	[41, 42]

[1] Sivaloganathan, D. M., Wan, X., Leon, G. & Brynildsen, M. P. Loss of Gre factors leads to phenotypic heterogeneity and cheating in *Escherichia coli* populations under nitric oxide stress. *mBio* **15**, e02229–24 (2024). URL `https://journals.asm.org/doi/10.1128/mbio.02229-24`.

[2] Bhattacharjee, T. & Datta, S. S. Confinement and activity regulate bacterial motion in porous media. *Soft Matter* **15**, 9920–9930 (2019). URL `http://xlink.rsc.org/?DOI=C9SM01735F`.

[3] Martínez-Calvo, A. *et al.* Morphological instability and roughening of growing 3D bacterial colonies. *Proceedings of the National Academy of Sciences* **119**, e2208019119 (2022). URL `https://pnas.org/doi/10.1073/pnas.2208019119`.

[4] Bhattacharjee, T. & Datta, S. S. Bacterial hopping and trapping in porous media. *Nature Communications* **10**, 2075 (2019). URL `http://www.nature.com/articles/s41467-019-10115-1`.

[5] Cordisco, E. & Serra, D. O. Moonlighting antibiotics: the extra job of modulating biofilm formation. *Trends in Microbiology* **33**, 459–471 (2025). URL `https://linkinghub.elsevier.com/retrieve/pii/S0966842X24003275`.

[6] Saltzman, W., Radomsky, M., Whaley, K. & Cone, R. Antibody diffusion in human cervical mucus. *Biophysical Journal* **66**, 508–515 (1994). URL `https://linkinghub.elsevier.com/retrieve/pii/S0006349594808021`.

[7] Casalini, T., Salvalaglio, M., Perale, G., Masi, M. & Cavallotti, C. Diffusion and Aggregation of Sodium Fluorescein in Aqueous Solutions. *The Journal of Physical Chemistry B* **115**, 12896–12904 (2011). URL `https://pubs.acs.org/doi/10.1021/jp207459k`.

[8] Radomsky, M. L., Whaley, K. J., Cone, R. A. & Saltzman, W. Macromolecules released from polymers: diffusion into unstirred fluids. *Biomaterials* **11**, 619–624 (1990). URL `https://linkinghub.elsevier.com/retrieve/pii/014296129090018L`.

[9] Andersson, D. I., Nicoloff, H. & Hjort, K. Mechanisms and clinical relevance of bacterial heteroresistance. *Nature Reviews Microbiology* **17**, 479–496 (2019). URL `https://www.nature.com/articles/s41579-019-0218-1`.

[10] El-Halfawy, O. M. & Valvano, M. A. Antimicrobial Heteroresistance: an Emerging Field in Need of Clarity. *Clinical Microbiology Reviews* **28**, 191–207 (2015). URL `https://journals.asm.org/doi/10.1128/CMR.00058-14`.

[11] Stein, W. D. *Channels, carriers, and pumps: an introduction to membrane transport* (Academic Press, San Diego, 1990).

[12] Bhattacharjee, T., Amchin, D. B., Ott, J. A., Kratz, F. & Datta, S. S. Chemotactic migration of bacteria in porous media. *Biophysical Journal* **120**, 3483–3497 (2021). URL `https://linkinghub.elsevier.com/retrieve/pii/S0006349521004276`.

[13] Bhattacharjee, T., Amchin, D. B., Alert, R., Ott, J. A. & Datta, S. S. Chemotactic smoothing of collective migration. *arXiv:2101.04576 [cond-mat, physics:nlin, physics:physics, q-bio]* (2021). URL `http://arxiv.org/abs/2101.04576`.

[14] Monod, J. The growth of bacterial cultures. *Annual Review of Microbiology* **3**, 371–394 (1949). URL https://www.annualreviews.org/doi/10.1146/annurev.mi.03.100149.002103.

[15] Scott, M., Klumpp, S., Mateescu, E. M. & Hwa, T. Emergence of robust growth laws from optimal regulation of ribosome synthesis. *Molecular Systems Biology* **10**, 747 (2014).

[16] Kovárová-Kovar, K. & Egli, T. Growth kinetics of suspended microbial cells: from single-substrate-controlled growth to mixed-substrate kinetics. *Microbiology and molecular biology reviews: MMBR* **62**, 646–666 (1998).

[17] Zhang, Y. *et al.* Persistent glucose consumption under antibiotic treatment protects bacterial community. *Nature Chemical Biology* **21**, 238–246 (2025). URL https://www.nature.com/articles/s41589-024-01708-z.

[18] Kim, K. *et al.* Mapping single-cell responses to population-level dynamics during antibiotic treatment. *Molecular Systems Biology* **19**, e11475 (2023). URL https://www.embopress.org/doi/10.15252/msb.202211475.

[19] Berryhill, B. A. *et al.* What's the Matter with MICs: Bacterial Nutrition, Limiting Resources, and Antibiotic Pharmacodynamics. *Microbiology Spectrum* **11**, e04091–22 (2023). URL https://journals.asm.org/doi/10.1128/spectrum.04091-22.

[20] Schink, S. J., Biselli, E., Ammar, C. & Gerland, U. Death rate of *E. coli* during starvation Is set by maintenance cost and biomass recycling. *Cell Systems* **9**, 64–73.e3 (2019). URL https://linkinghub.elsevier.com/retrieve/pii/S240547121930198X.

[21] Horii, T., Kimura, T., Sato, K., Shibayama, K. & Ohta, M. Emergence of Fosfomycin-Resistant Isolates of Shiga-Like Toxin-Producing *Escherichia coli* O26. *Antimicrobial Agents and Chemotherapy* **43**, 789–793 (1999). URL https://journals.asm.org/doi/10.1128/AAC.43.4.789.

[22] Silver, L. L. Fosfomycin: Mechanism and Resistance. *Cold Spring Harbor Perspectives in Medicine* **7**, a025262 (2017). URL http://perspectivesinmedicine.cshlp.org/lookup/doi/10.1101/cshperspect.a025262.

[23] Ishihama, Y. *et al.* Protein abundance profiling of the Escherichia coli cytosol. *BMC Genomics* **9**, 102 (2008). URL http://bmcgenomics.biomedcentral.com/articles/10.1186/1471-2164-9-102.

[24] Hancock, V., Ferrières, L. & Klemm, P. Biofilm formation by asymptomatic and virulent urinary tract infectious *Escherichia coli* strains. *FEMS Microbiology Letters* **267**, 30–37 (2007). URL https://academic.oup.com/femsle/article-lookup/doi/10.1111/j.1574-6968.2006.00507.x.

[25] Feist, A. M. *et al.* A genome-scale metabolic reconstruction for *Escherichia coli* K-12 MG1655 that accounts for 1260 ORFs and thermodynamic information. *Molecular Systems Biology* **3**, 121 (2007). URL https://www.embopress.org/doi/10.1038/msb4100155.

[26] Biselli, E., Schink, S. J. & Gerland, U. Slower growth of *Escherichia coli* leads to longer survival in carbon starvation due to a decrease in the maintenance rate. *Molecular Systems Biology* **16** (2020). URL https://onlinelibrary.wiley.com/doi/10.15252/msb.20209478.

[27] Senn, H., Lendenmann, U., Snozzi, M., Hamer, G. & Egli, T. The growth of Escherichia coli in glucose-limited chemostat cultures: a re-examination of the kinetics. *Biochimica et Biophysica Acta (BBA) - General Subjects* **1201**, 424–436 (1994). URL https://linkinghub.elsevier.com/retrieve/pii/0304416594900728.

[28] Hermsen, R., Okano, H., You, C., Werner, N. & Hwa, T. A growth-rate composition formula for the growth of *E. coli* on co-utilized carbon substrates. *Molecular Systems Biology* **11**, 801 (2015). URL https://www.embopress.org/doi/10.15252/msb.20145537.

[29] Karlowsky, J. A. *et al.* Susceptibility of Clinical Isolates of Escherichia coli to Fosfomycin as Measured by Four *In Vitro* Testing Methods. *Journal of Clinical Microbiology* **58**, e01306–20 (2020). URL https://journals.asm.org/doi/10.1128/JCM.01306-20.

[30] Cronenberg, C. & Van Den Heuvel, J. Determination of glucose diffusion coefficients in biofilms with microelectrodes. *Biosensors and Bioelectronics* **6**, 255–262 (1991). URL https://linkinghub.elsevier.com/retrieve/pii/095656639180011L.

[31] Bashkatov, A. N. *et al.* Glucose and Mannitol Diffusion in Human Dura Mater. *Biophysical Journal* **85**, 3310–3318 (2003). URL https://linkinghub.elsevier.com/retrieve/pii/S000634950374750X.

[32] Stewart, P. S. Diffusion in Biofilms. *Journal of Bacteriology* **185**, 1485–1491 (2003). URL https://journals.asm.org/doi/10.1128/JB.185.5.1485-1491.2003.

[33] Meulemans, A., Paycha, F., Hannoun, P. & Vulpillat, M. Measurement and clinical and pharmacokinetic implications of diffusion coefficients of antibiotics in tissues. *Antimicrobial Agents and Chemotherapy* **33**, 1286–1290 (1989). URL https://journals.asm.org/doi/10.1128/AAC.33.8.1286.

[34] Lehmenkühler, A., Kersting, U. & Nicholson, C. Diffusion of penicillin in agar and cerebral cortex of the rat. *Brain Research* **444**, 181–183 (1988). URL https://linkinghub.elsevier.com/retrieve/pii/0006899388909262.

[35] Sharma, D., Misba, L. & Khan, A. U. Antibiotics versus biofilm: an emerging battleground in microbial communities. *Antimicrobial Resistance & Infection Control* **8**, 76 (2019). URL https://aricjournal.biomedcentral.com/articles/10.1186/s13756-019-0533-3.

[36] Moore-Ott, J. A., Chiu, S., Amchin, D. B., Bhattacharjee, T. & Datta, S. S. A biophysical threshold for biofilm formation. *eLife* **11**, e76380 (2022). URL https://elifesciences.org/articles/76380.

[37] Kirby, A. E., Garner, K. & Levin, B. R. The Relative Contributions of Physical Structure and Cell Density to the Antibiotic Susceptibility of Bacteria in Biofilms. *Antimicrobial Agents and Chemotherapy* **56**, 2967–2975 (2012). URL https://journals.asm.org/doi/10.1128/AAC.06480-11.

[38] Zhang, Q., Zhao, G., Yang, N. & Zhang, L. Fasting blood glucose levels in patients with different types of diseases. In *Progress in Molecular Biology and Translational Science*, vol. 162, 277–292 (Elsevier, 2019). URL https://linkinghub.elsevier.com/retrieve/pii/S1877117319300158.

[39] Cowart, S. L. & Stachura, M. E. Glucosuria. In Walker, H. K., Hall, W. D. & Hurst, J. W. (eds.) *Clinical Methods: The History, Physical, and Laboratory Examinations* (Butterworths, Boston, 1990), 3rd edn. URL `http://www.ncbi.nlm.nih.gov/books/NBK245/`.

[40] Ferraris, R. P., Yasharpour, S., Lloyd, K. C., Mirzayan, R. & Diamond, J. M. Luminal glucose concentrations in the gut under normal conditions. *American Journal of Physiology-Gastrointestinal and Liver Physiology* **259**, G822–G837 (1990). URL `https://www.physiology.org/doi/10.1152/ajpgi.1990.259.5.G822`.

[41] Kuiper, S. G. *et al.* Pharmacokinetics of fosfomycin in patients with prophylactic treatment for recurrent *Escherichia coli* urinary tract infection. *Journal of Antimicrobial Chemotherapy* **75**, 3278–3285 (2020). URL `https://academic.oup.com/jac/article/75/11/3278/5876615`.

[42] Di Stefano, A. F. D. *et al.* Fosfomycin Pharmacokinetic Profile in Plasma and Urine and Quantitative Estimation in Prostate and Seminal Vesicles after One and Two Consecutive Doses of Oral Fosfomycin Trometamol in Healthy Male Volunteers. *Antibiotics* **11**, 1458 (2022). URL `https://www.mdpi.com/2079-6382/11/11/1458`.

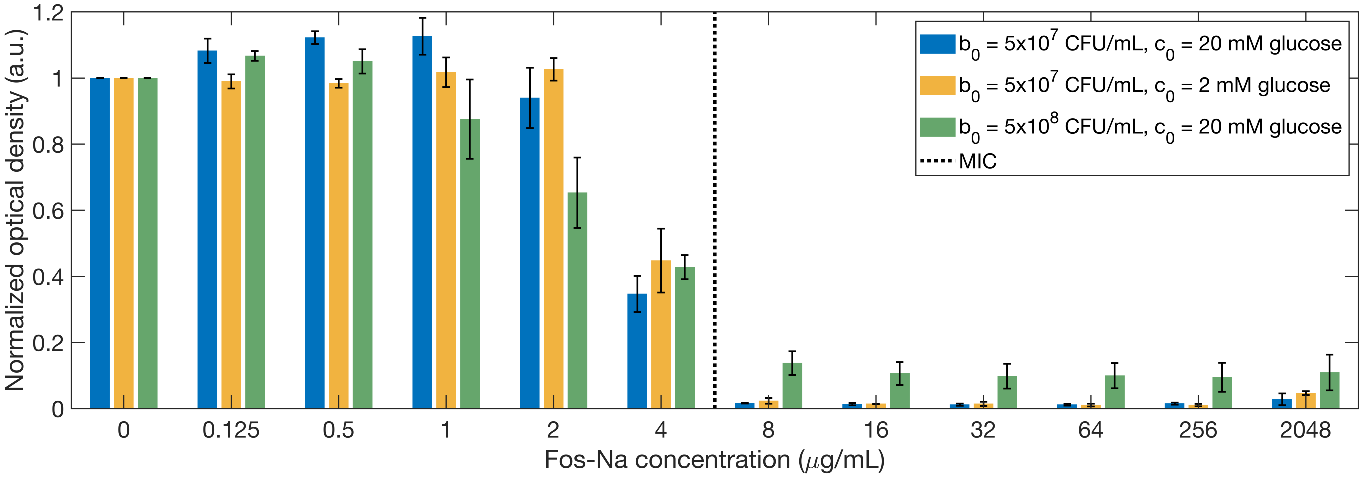

FIG. S1: Minimum inhibitory concentration (MIC) of fosfomycin evaluated across varying glucose concentrations and cell densities. MIC (dashed line) is defined as the concentration above which no growth, measured by optical density (OD) of liquid cultures, is detected across conditions. The base condition (blue bars) contains a starting density of 5×10^7 CFU/mL growing in 20 mM glucose. With fewer nutrients (yellow bars, starting cell density of 5×10^7 CFU/mL growing in 2 mM glucose) or more cells (green bars, starting cell density of 5×10^8 CFU/mL growing in 20 mM glucose), the MIC remains the same. When starting with a higher cell density (green bars), the high antibiotic ODs (OD > 8 μg/mL) are higher than low cell density cases due to the large initial biomass present in each microplate well. For all conditions, normalized optical density is the optical density after 12 hours of antibiotic exposure normalized to the antibiotic free condition. Each bar represents a mean of 4 biological replicates and error bars indicate standard deviation. Source data are provided as a Source Data file.

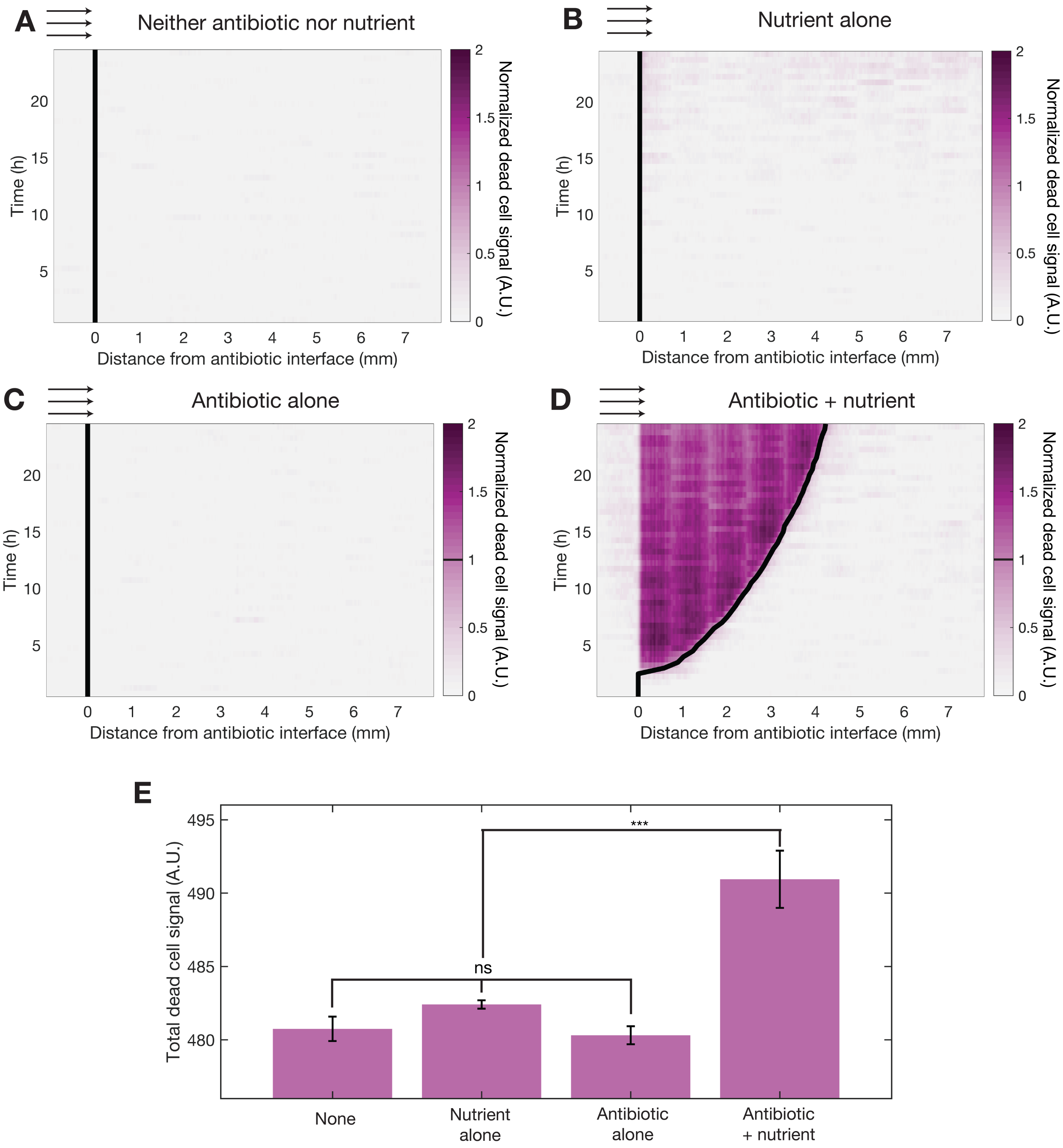

FIG. S2: Propidium iodide (PI) dead-cell fluorescent signal from 1 replicate of time-lapse confocal microscopy across conditions. For each condition, normalized dead cell signal is obtained by subtracting a baseline noise value and then dividing by an arbitrary threshold value, both of which are constant across conditions. Then, death front position (black line) is defined as the furthest x position with a normalized signal value of 1. **A** Kymograph of PI signal for 10^8 CFU/mL stationary phase *E. coli* population. No nutrients or antibiotic are placed in the reservoir. **B** Kymograph of PI signal for 10^8 CFU/mL stationary phase *E. coli* population encountering a diffusing source of 22.2 mM glucose and no antibiotic. **C** Kymograph of PI signal for 10^8 CFU/mL stationary phase *E. coli* population encountering a diffusing source of 2048 μg/mL Fos-Na and no glucose, the same condition seen in Main Text Fig. 1C and Supplementary Movie 1. **D** Kymograph of PI signal for 10^8 CFU/mL stationary phase *E. coli* population encountering a diffusing source of 2048 μg/mL Fos-Na and 0.22 mM glucose, the same condition seen in Main Text Fig. 1D and Supplementary Movie 2. **E** Total dead cell fluorescence signal for each condition **A-D** averaged across 3 biological replicates with standard deviation shown by error bars. Statistical significance is assessed using a 1-way ANOVA test; *** indicates $p < 0.001$ and ns indicates a difference that is not significant. Thus, antibiotic alone does not cause appreciable cell death compared to nutrient rich or nutrient poor environments without antibiotic. Source data are provided as a Source Data file.

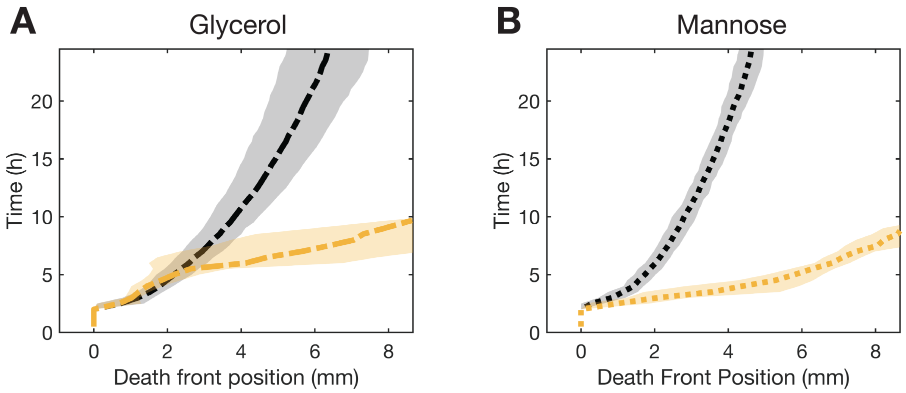

FIG. S3: Increasing nutrient source concentration from 0.22 mM (black line) to 2.2 mM (yellow line) **A** glycerol and **B** mannose increases death front clearance speed for a 10^8 CFU/mL *E. coli* population treated with 2048 μg/mL Fos-Na. Shading around all lines represents standard deviation in death front position at each time point across 3 biological replicates. Source data are provided as a Source Data file.

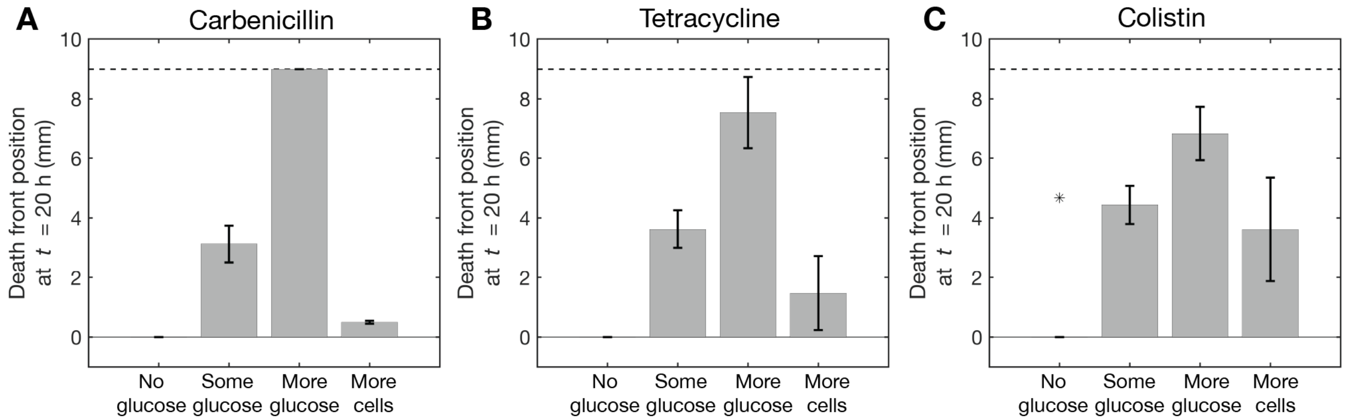

FIG. S4: Nutrient availability shapes clearance of structured bacterial populations by **A** carbenicillin, **B** tetracycline, and **C** colistin. **A** Carbenicillin is supplied at a concentration of 4096 μg/mL, or 256× the MIC of carbenicillin (Supplementary Fig. 5A), across conditions. **B** Tetracycline is supplied at a concentration of 256 μg/mL, or 256× the MIC of tetracycline (Supplementary Fig. 5B), across conditions. **C** Colistin is supplied at a concentration of 1024 μg/mL, or 256× the MIC of colistin (Supplementary Fig. 5C), across conditions. For **A-C**, "no glucose" condition represents clearance of a 10^8 CFU/mL stationary phase *E. coli* population encountering a diffusing source of antibiotic and no glucose. "Some glucose" condition represents clearance of a 10^8 CFU/mL stationary phase *E. coli* population encountering a diffusing source of antibiotic and 2.2 mM glucose. "More glucose" condition represents clearance of a 10^8 CFU/mL stationary phase *E. coli* population encountering a diffusing source of antibiotic and 22.2 mM glucose. "More cells" condition represents clearance of a 10^9 CFU/mL stationary phase *E. coli* population encountering a diffusing source of antibiotic and 2.2 mM glucose. Death front position each condition is evaluated after $t = 20$ h by propidium iodide fluorescence signal exceeding a threshold value for each antibiotic. When no cell death is detected anywhere in the population, a death front position of 0 mm is reported. For **A-B**, each bar represents the mean of 3 biological replicates and error bars indicate standard deviation. For **C**, each bar represents the mean of 5 biological replicates and error bars indicate standard deviation. Asterisk in "no glucose" condition represents an outlier of detected death front position from 1 of 5 replicates. Source data are provided as a Source Data file.

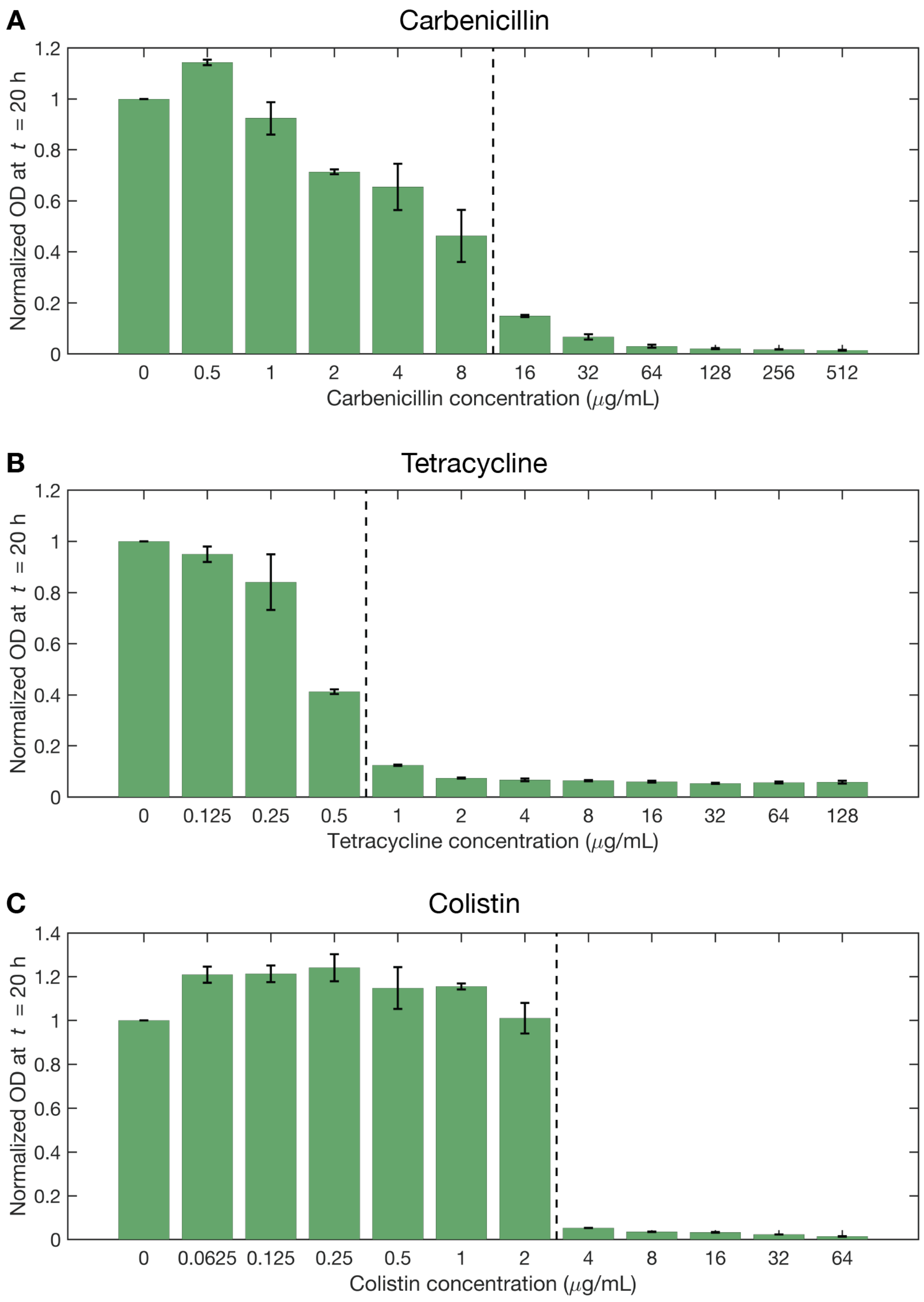

FIG. S5: Minimum inhibitory concentration (MIC) of **A** carbenicillin, **B** tetracycline, and **C** colistin. MIC (dashed line) for each antibiotic is defined as the concentration above which no substantial growth, measured by optical density (OD) of liquid cultures, is detected. For **A-C**, MIC is evaluated for a starting cell density of 10^7 CFU/mL grown in M9 media with 20 mM glucose. Normalized optical density is the optical density after 20 hours of antibiotic exposure normalized to the antibiotic free condition. Each bar represents a mean of 2 biological replicates and error bars indicate standard deviation. Source data are provided as a Source Data file.

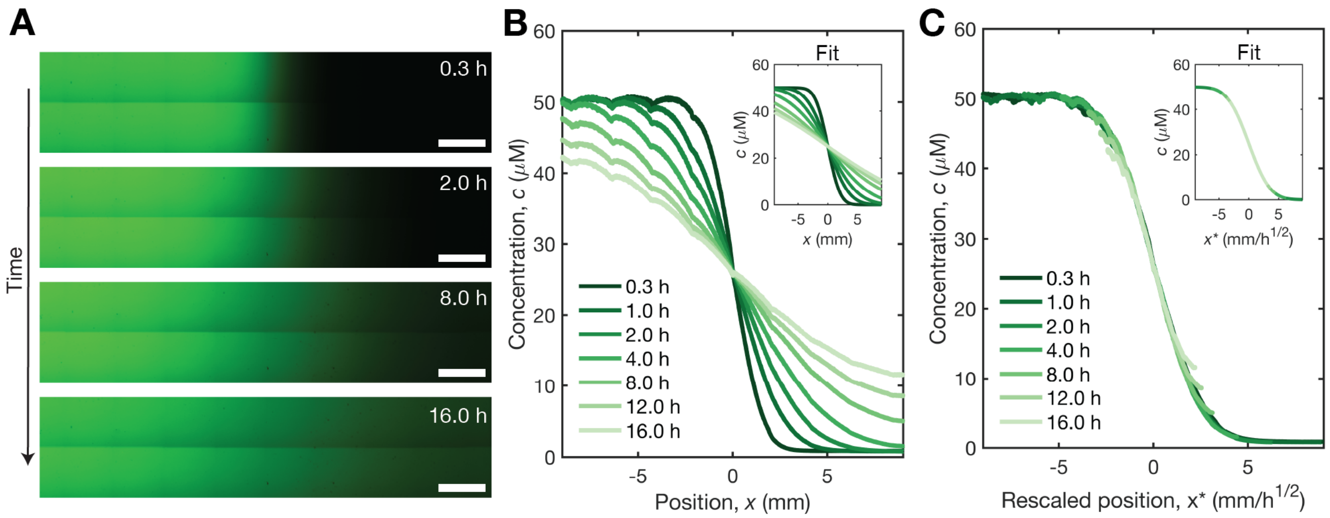

FIG. S6: Fluorescein, a representative and fluorescent small molecule, transport through the granular hydrogel matrix is entirely diffusive over time. **A** Select images of fluorescein diffusion in the granular hydrogel matrix over time. Initially, we pattern the granular hydrogel matrices, which are swollen in the same M9 liquid media for cell based experiments, to mimic diffusion of a solute between two semi-infinite planar domains. That is, on one half of the dish we had a source of fluorescein at an initial concentration of $c_1 = 50$ μM. At $t = 0$ h, this source concentration diffuses into a reservoir initially at $c_2 = 0$ μM. Scale bar is 1 mm. **B** We visualize the imaging signal in space, a proxy for fluorescein concentration, from one replicate over time. **C** We confirm a fully diffusive profile by collapsing the data across all $x^* \equiv \frac{x}{\sqrt{t}}$. Insets in **B and C** include the fit of the analytical solution for diffusion between two semi-infinite planar domains, $c(x,t) = (\frac{c_1+c_2}{2}) - (\frac{c_1+c_2}{2})\mathrm{erf}(\frac{x}{2D\sqrt{t}})$ where $\mathrm{erf}(x) = \frac{2}{\pi}\int_0^x -y^2\, dy$ to obtain an estimate of the diffusion coefficient, $D = 2.2 \pm 0.1$ mm^2/h. Source data are provided as a Source Data file.

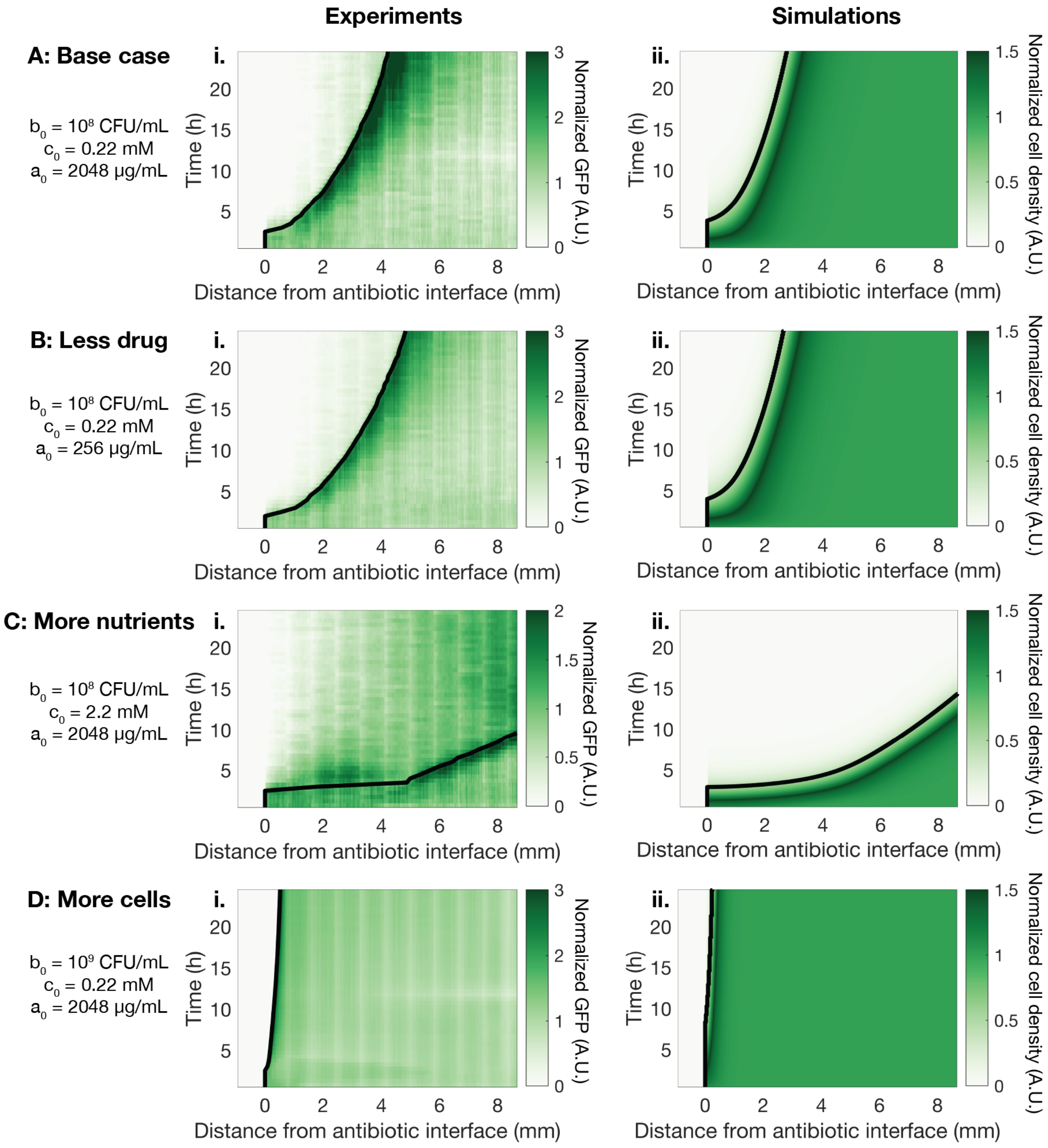

FIG. S7: Qualitative comparison between GFP fluorescence signal from experiments (**i**) and simulation cell density (**ii**). **A** One experimental replicate and simulations for a 10^8 CFU/mL *E. coli* population encountering a diffusing source of 0.22 mM glucose and 2048 μg/mL Fos-Na. **B** One experimental replicate and simulations for a 10^8 CFU/mL *E. coli* population encountering a diffusing source of 0.22 mM glucose and 256 μg/mL Fos-Na. **C** One experimental replicate and simulations for a 10^8 CFU/mL *E. coli* population encountering a diffusing source of 2.2 mM glucose and 2048 μg/mL Fos-Na. **D** One experimental replicate and simulations for a 10^9 CFU/mL *E. coli* population encountering a diffusing source of 0.22 mM glucose and 2048 μg/mL Fos-Na. For **A-D**, experimental GFP signal is normalized by blank subtracting each value by the average signal in the cell free region at each time point then dividing by the average signal in the cell rich region at $t = 0.5$ h. Simulation cell density is normalized by the initial cell density. Source data are provided as a Source Data file.

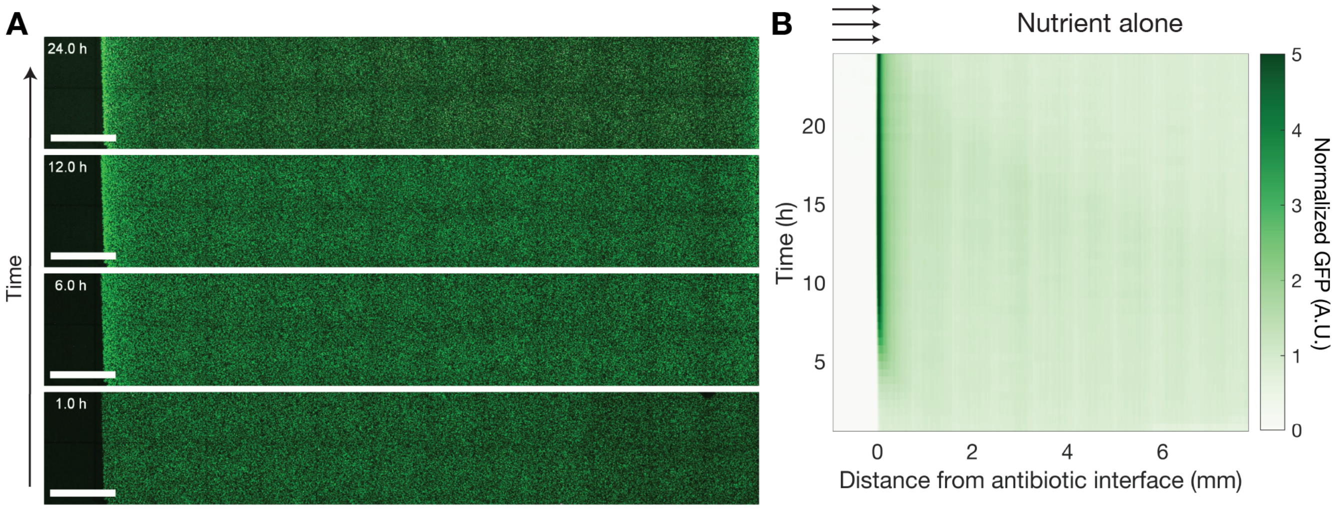

FIG. S8: High nutrient, no antibiotic conditions confirm cells remain immotile within granular hydrogel matrices. **A** Select images of one replicate of 10^8 CFU/mL stationary phase *E. coli* population encountering a diffusing source of 22.2 mM glucose, demonstrating surface growth near the nutrient interface. Scale bar is 1 mm. **B** Kymograph of GFP signal from time course experiments with 10^8 CFU/mL stationary phase *E. coli* population encountering a diffusing source of 22.2 mM glucose. Normalized GFP signal is achieved by blank subtracting each value by the average signal in the cell free region over time, dividing by the average signal in the cell rich region at $t = 0.5$ h, and then averaging across 3 biological replicates. No chemotactic wave is detected into the cell-free, nutrient-rich region confirming cells remain immotile in the granular hydrogel matrix, even in high nutrient environments without antibiotics. Source data are provided as a Source Data file.

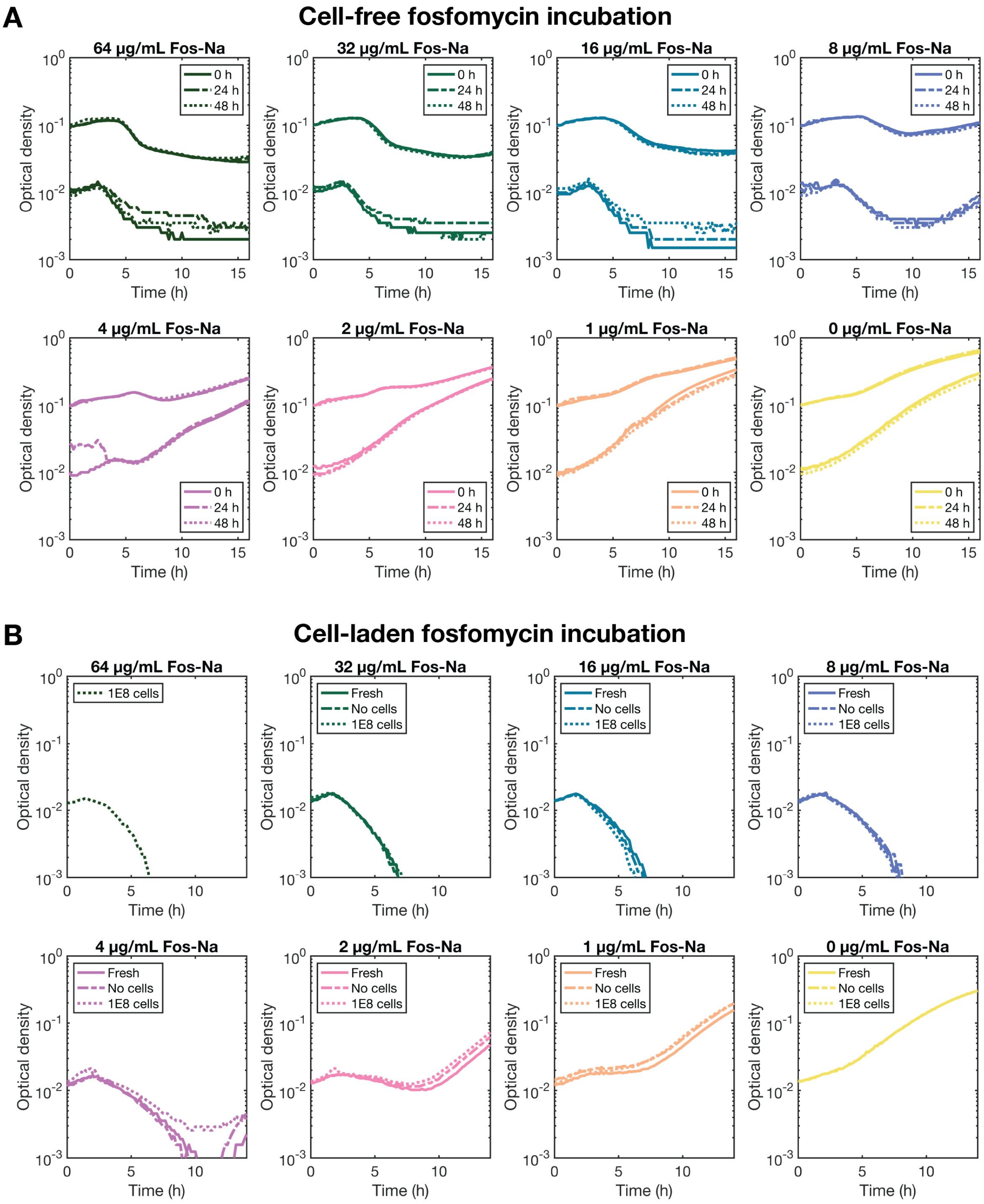

FIG. S9: Growth and death curves of cells with fresh vs. pre-incubated antibiotic. In **A**, the legend indicates the time antibiotic stock was pre-incubated at 37°C prior to the experiment, in the absence of any cells. Then, 2 initial cell densities are incubated in fresh M9 media with 22 mM glucose at varying concentrations of antibiotic from each fosfomycin stock. Each line represents mean growth of two replicates. In **B**, fosfomycin was pre-incubated with an initially 10^8 CFU/mL cell population in M9 media with 22 mM glucose at 37°C for 24 hours. Then, one initial cell density is incubated in an equal volume mix of pre-digested M9 22 mM glucose media and fresh M9 22 mM glucose media with either freshly made fosfomycin, cell-free pre-incubated fosfomycin, or cell-laden pre-incubated fosfomycin, as indicated by the legend. Each line represents mean growth of three replicates. In **A** and **B**, growth and death does not vary across conditions suggesting that fosfomycin does not degrade significantly over the time scale of experiments in the presence or absence of cells. We note that growth dynamics change very slightly with the inclusion of pre-digested media compare to fresh media for all conditions at near-MIC concentrations likely due to the presence of waste products from the pre-digested media. Source data are provided as a Source Data file.

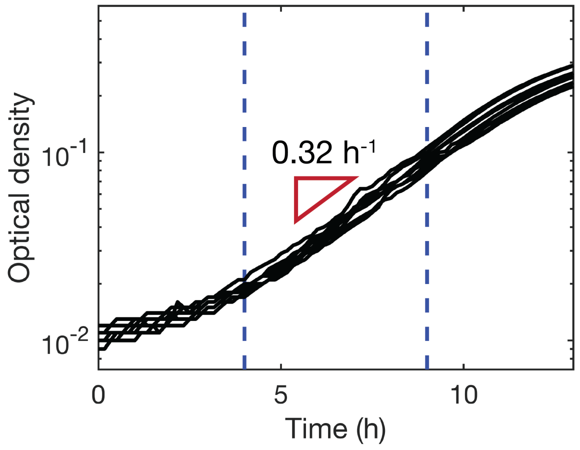

FIG. S10: Growth of *E. coli* in 20.0 mM glucose M9 media. Black lines show 8 replicates across 4 independent experiments, each of which was fit with an exponential curve between 4-9 h (blue dotted lines), to yield an average exponential growth rate of 0.32 h^{-1}. Source data are provided as a Source Data file.

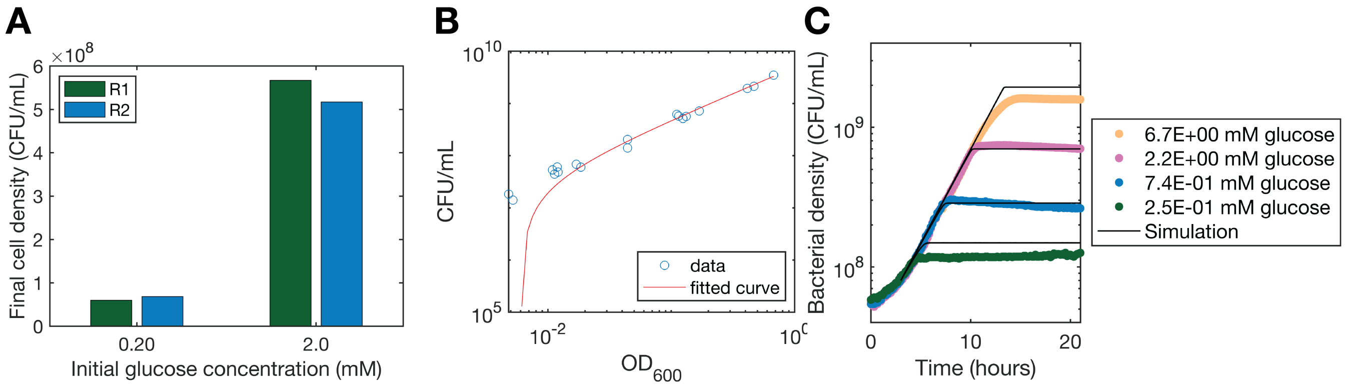

FIG. S11: Experiments to parameterize κ. **A** Final cell density of two replicate cultures inoculated with $< 10^7$ CFU/mL. Final yield was used to calculate the nutrient consumption rate per cell, κ according to the expression $\kappa = \frac{c_0 g}{b_f} = 2.0 \times 10^{-11} \pm 2 \times 10^{-12}$ (mM)(CFU/mL)$^{-1}$(h)$^{-1}$. **B** OD_{600} to CFU/mL linear fit conversion from measurements across many independent experiments. **C** Good agreement between experimental growth curves (dots) and well-mixed simulations (black lines) across 4 nutrient conditions confirms an appropriate value for κ. Experimental measurements were converted from OD_{600} to CFU/mL according to the linear fit in **B**. Source data are provided as a Source Data file.

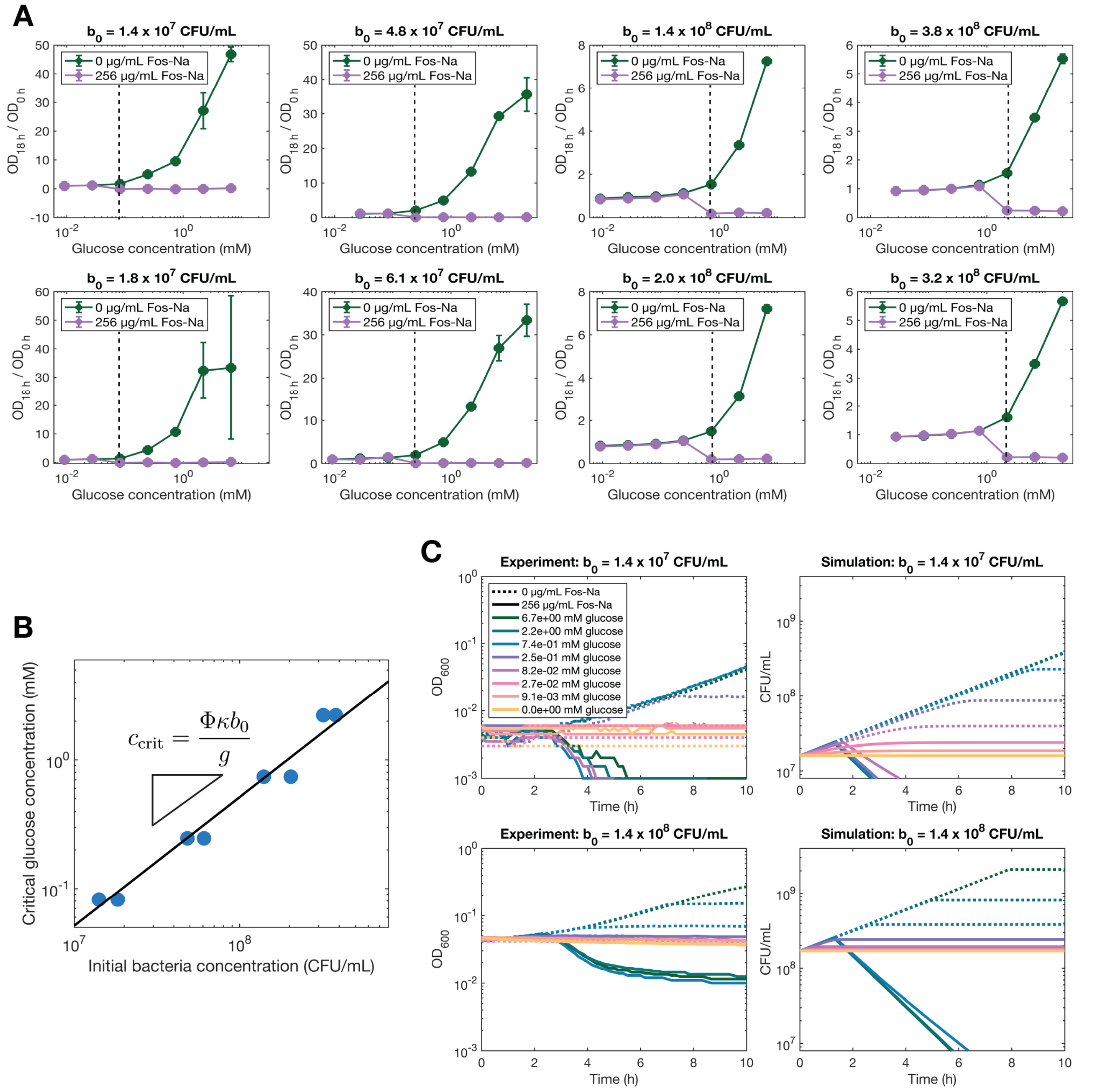

FIG. S12: Experiments to parameterize growth threshold parameter Φ. **A** Optical density after 18 h growth compared to initial optical density for varying inoculum densities cultured in a range of glucose concentrations. At and above a threshold nutrient concentration, c_{crit}, marked by a dashed line on each plot, cells died in the presence of 256 µg/mL Fos-Na. Each point is a mean of two technical replicates with error bars to indicate the standard deviation. Example time course data for two experiments in **A** are shown in panel **C**. **B** The critical glucose concentration c_{crit} varies linearly with initial cell density b_0 as expected from our theory. Each blue dot corresponds to an independent experiment shown in **A** and the black line follows the equation $c_{\text{crit}} = \frac{\Phi \kappa b_0}{g}$, where $\Phi = 1.5 \pm 0.3$ averaged across all 8 experiments. **C** Simulating well-mixed growth with the measured value of Φ gives qualitatively similar time course results to experiments. Source data are provided as a Source Data file.

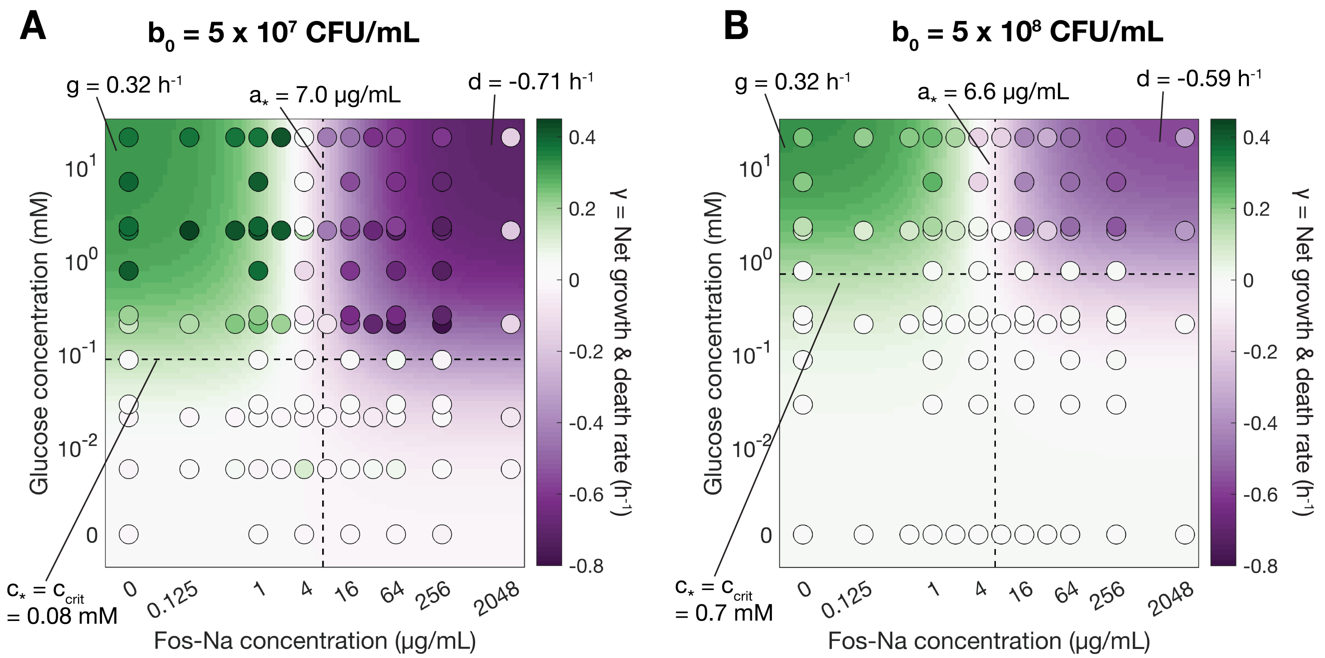

FIG. S13: Experiments to parameterize a_* and d. We measure net cell growth and death rate γ as a function of nutrient and antibiotic concentration for **A** low and **B** high initial cell densities. Dots are experimentally measured rates from UV-vis experiments across nutrient, antibiotic, and cell density conditions. Data represents over 100 conditions and each dot is the mean of 2-4 replicates. Background color is the fit for the function $\gamma = \left(\frac{c}{c+c_*}\right)\left(\frac{(g+d)a_*}{a+a_*} - d\right)$ where g is fixed to 0.32 h^{-1}. Simulations use fit parameters from the low cell density conditions **A**, as summarized in Supplementary Table 1. Source data are provided as a Source Data file.

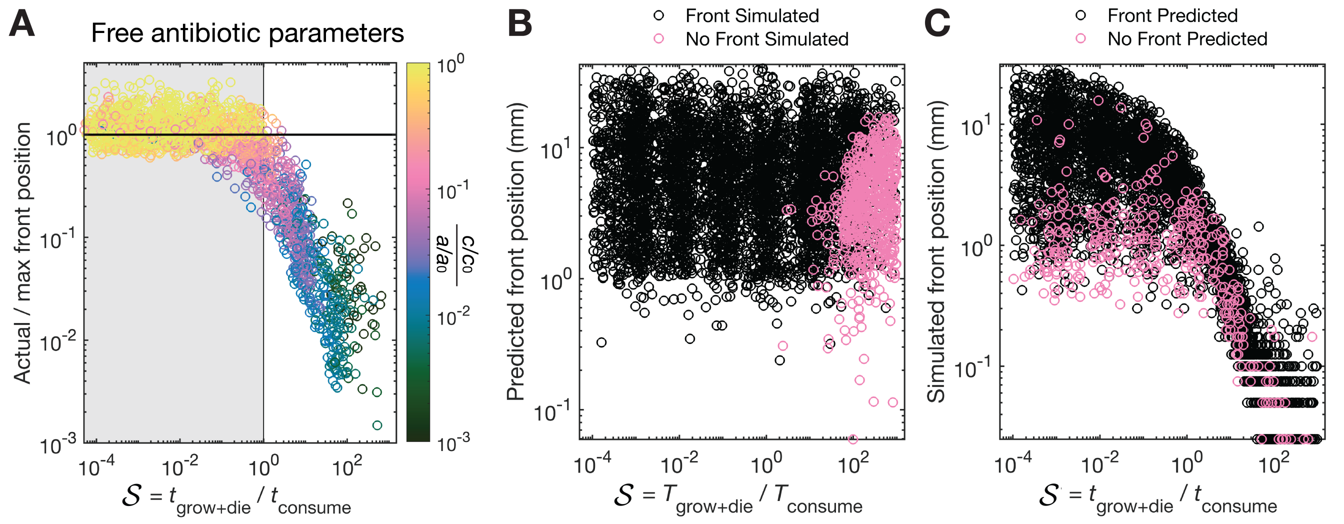

FIG. S14: **A** Sweeping across all parameter ranges, without constraining $D_c = D_a$ and $c_0/c_* = a_0/a_*$, as in Main Text Fig. 4C, the actual simulated front position still collapses away from the theoretical maximum value when the timescale for growth and death $t_{\text{grow+die}}$ is much larger (slower) than the timescale for nutrient consumption t_{consume}. Each simulation is evaluated 12 h after a death front is detected and cases where no front forms in the predicted time window are excluded. Point coloring describes the normalized nutrient concentration at the front position vs. the normalized antibiotic concentration at the front. **B** For certain simulations across all presented conditions, no front is simulated (pink points) even when one is predicted. These are concentrated to large $\mathcal{S}$ and can occur across the range of all predicted front positions (black points). **C** Similarly, for some simulation conditions, no front is predicted to form (pink points) yet a front is simulated. These outlier cases occur across all values of $\mathcal{S}$ and represent a small fraction of total simulations where a front is successfully predicted. Source data are provided as a Source Data file.

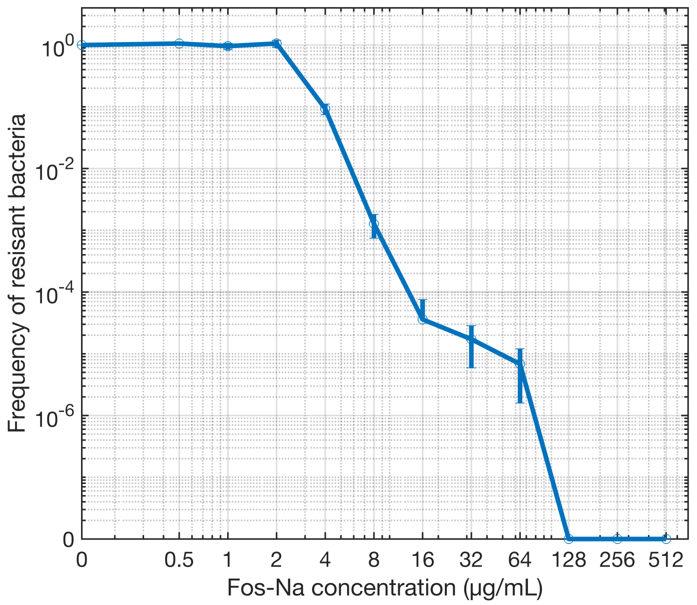

FIG. S15: Population analysis profiling of cell strain used in this paper reveals heteroresistance to fosfomycin. CFU counts for cells that grew on at each antibiotic concentration are compared to antibiotic-free CFU counts to obtain the frequency of resistant bacteria. Since resistant subpopulations with frequencies greater than 10^{-7} are detected over an 8-fold concentration range of fosfomycin our cells can be defined as heteroresistant [9]. The frequency detection threshold for the assay is 10^{-8} and antibiotic concentrations where no cells are detected are marked with a frequency of zero. Each dot represents the mean of 3 biological replicates with standard deviation shown as error bars. Source data are provided as a Source Data file.

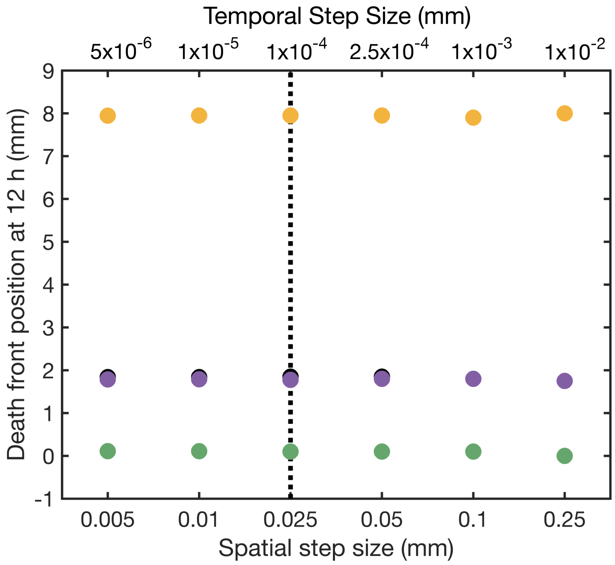

FIG. S16: Simulations results do not change appreciably for varying varying spatial and temporal discretization. To assess the sensitivity of our results to numerical discretization, we repeat four representative simulations at varying discretizations. Each color dot represents a different set of initial conditions for the representative simulations. Black is the base case with $b_0 = 10^8$ CFU/mL, $c_0 = 2.22$ mM, $a_0 = 2048$ µg/mL Fos-Na. Purple has less antibiotic with $a_0 = 256$ µg/mL Fos-Na. Yellow has more nutrient with $c_0 = 0.22$ mM glucose. Green has more cells with $b_0 = 10^9$ CFU/mL. Dashed line denotes discretization used in the numerical simulations across the paper. The death front position after 12 h obtained from the simulations is not strongly sensitive to the choice of numerical discretization for spatial and temporal steps smaller than those used for the simulations presented in the main text (dashed line). Thus, our choice of discretization is sufficiently finely resolved such that the results in the numerical simulations are not appreciably influenced by discretization. Source data are provided as a Source Data file.
