# Supplementary material for: A nutrient bottleneck controls antibiotic efficacy in structured bacterial populations
Source: Nat Commun. 2026 Feb 20;17:3337. doi: 10.1038/s41467-026-69625-4 (PMC13066622; doi:10.1038/s41467-026-69625-4)
Supplement: Supplementary file 2 — Description of Additional Supplementary Files [file 41467_2026_69625_MOESM2_ESM.pdf]

Title: Supplementary Movie 1

Description: Time-lapse microscopy of a 108 CFU/mL cell population being treated with 2048 µg/mL Fos-Na and 0 mM glucose. Green GFP signal corresponds to live cells with intact cell membranes. Magenta signal is propidium iodide, a dead cell indicator. Three biological replicates are shown and scale bar is 1 mm.

Title: Supplementary Movie 2

Description: Time-lapse microscopy of a 108 CFU/mL cell population being treated with 2048 µg/mL Fos-Na and 0.22 mM glucose. Green GFP signal corresponds to live cells with intact cell membranes. Magenta signal is propidium iodide, a dead cell indicator. Three biological replicates are shown and scale bar is 1 mm.

Title: Supplementary Movie 3

Description: Time-lapse microscopy of a 108 CFU/mL cell population being treated with 256 µg/mL Fos-Na and 0.22 mM glucose. Green GFP signal corresponds to live cells with intact cell membranes. Magenta signal is propidium iodide, a dead cell indicator. Three biological replicates are shown and scale bar is 1 mm.

Title: Supplementary Movie 4

Description: Time-lapse microscopy of a 108 CFU/mL cell population being treated with 2048 µg/mL Fos-Na and 2.2 mM glucose. Green GFP signal corresponds to live cells with intact cell membranes. Magenta signal is propidium iodide, a dead cell indicator. Three biological replicates are shown and scale bar is 1 mm.

Title: Supplementary Movie 5

Description: Time-lapse microscopy of a 109 CFU/mL cell population being treated with 2048 µg/mL Fos-Na and 0.22 mM glucose. Green GFP signal corresponds to live cells with intact cell membranes. Magenta signal is propidium iodide, a dead cell indicator. Three biological replicates are shown and scale bar is 1 mm.

Title: Supplementary Movie 6

Description: Numerical simulation of a 108 CFU/mL cell population being treated with 2048 µg/mL Fos-Na and 0.22 mM glucose. Y axis is normalized by initial concentration of cells, nutrient, and antibiotic.

Title: Supplementary Movie 7

Description: Time-lapse microscopy of a 108 CFU/mL cell population being treated with 64 µg/mL Fos-Na and 2.2 mM glucose. Green GFP signal corresponds to live cells with intact cell membranes. Magenta signal is propidium iodide, a dead cell indicator. Three biological replicates are shown and scale bar is 1 mm.

Title: Supplementary Movie 8

Description: Time-lapse microscopy of a 108 CFU/mL cell population being treated with 16 μg/mL Fos-Na and 2.2 mM glucose. Green GFP signal corresponds to live cells with intact cell membranes. Magenta signal is propidium iodide, a dead cell indicator. Three biological replicates are shown and scale bar is 1 mm.

Title: Supplementary Movie 9

Time-lapse microscopy of a 109 CFU/mL cell population
