# Supplementary material for: A nutrient bottleneck controls antibiotic efficacy in structured bacterial populations
Source: Nat Commun. 2026 Feb 20;17:3337. doi: 10.1038/s41467-026-69625-4 (PMC13066622; doi:10.1038/s41467-026-69625-4)
Supplement: Supplementary file 12 — Reporting Summary [file 41467_2026_69625_MOESM12_ESM.pdf]

nature portfolio

Corresponding author(s): Sujit Datta
Last updated by author(s): Jan 7, 2026

Reporting Summary

Nature Portfolio wishes to improve the reproducibility of the work that we publish. This form provides structure for consistency and transparency in reporting. For further information on Nature Portfolio policies, see our Editorial Policies and the Editorial Policy Checklist.

Statistics

For all statistical analyses, confirm that the following items are present in the figure legend, table legend, main text, or Methods section.

n/a	Confirmed	
☐	☒	The exact sample size (*n*) for each experimental group/condition, given as a discrete number and unit of measurement
☐	☒	A statement on whether measurements were taken from distinct samples or whether the same sample was measured repeatedly
☐	☒	The statistical test(s) used AND whether they are one- or two-sided<br>*Only common tests should be described solely by name; describe more complex techniques in the Methods section.*
☒	☐	A description of all covariates tested
☒	☐	A description of any assumptions or corrections, such as tests of normality and adjustment for multiple comparisons
☐	☒	A full description of the statistical parameters including central tendency (e.g. means) or other basic estimates (e.g. regression coefficient) AND variation (e.g. standard deviation) or associated estimates of uncertainty (e.g. confidence intervals)
☐	☒	For null hypothesis testing, the test statistic (e.g. *F*, *t*, *r*) with confidence intervals, effect sizes, degrees of freedom and *P* value noted<br>*Give P values as exact values whenever suitable.*
☒	☐	For Bayesian analysis, information on the choice of priors and Markov chain Monte Carlo settings
☒	☐	For hierarchical and complex designs, identification of the appropriate level for tests and full reporting of outcomes
☒	☐	Estimates of effect sizes (e.g. Cohen's *d*, Pearson's *r*), indicating how they were calculated

Our web collection on statistics for biologists contains articles on many of the points above.

Software and code

Policy information about availability of computer code

Data collection	Imaging data was collected on either a Nikon AXR or A1 inverted laser scanning confocal microscope with its supporting software. Optical density data was collected using a Biotek Epoch2 plate reader and its supporting software.
Data analysis	All data analysis and simulations were conducted using MATLAB 2024A. The code generated during the current study are available at Code Ocean at DOI: 10.24433/CO.4205040.v1.

For manuscripts utilizing custom algorithms or software that are central to the research but not yet described in published literature, software must be made available to editors and reviewers. We strongly encourage code deposition in a community repository (e.g. GitHub). See the Nature Portfolio guidelines for submitting code & software for further information.

Data

Policy information about availability of data

All manuscripts must include a data availability statement. This statement should provide the following information, where applicable:

- Accession codes, unique identifiers, or web links for publicly available datasets
- A description of any restrictions on data availability
- For clinical datasets or third party data, please ensure that the statement adheres to our policy

The compressed imaging data generated in this study are provided in the Supplementary Movie files on Zenodo https://zenodo.org/records/14990206. Additional experimental and simulation data generated in this study are provided in the Source Data file.

Research involving human participants, their data, or biological material

Policy information about studies with human participants or human data. See also policy information about sex, gender (identity/presentation), and sexual orientation and race, ethnicity and racism.

Reporting on sex and gender	N/A
Reporting on race, ethnicity, or other socially relevant groupings	N/A
Population characteristics	N/A
Recruitment	N/A
Ethics oversight	N/A

Note that full information on the approval of the study protocol must also be provided in the manuscript.

Field-specific reporting

Please select the one below that is the best fit for your research. If you are not sure, read the appropriate sections before making your selection.

[x] Life sciences [ ] Behavioural & social sciences [ ] Ecological, evolutionary & environmental sciences

For a reference copy of the document with all sections, see nature.com/documents/nr-reporting-summary-flat.pdf

Life sciences study design

All studies must disclose on these points even when the disclosure is negative.

Sample size	No formal statistical power calculations were performed to predetermine sample sizes. Instead, sample sizes were chosen a priori based on established experimental norms in biophysics and microbiology where independent biological replicates with n = 3 are standard practice for mechanistic and quantitative measurements. Thus, each imaging experiment was performed using at least three independent biological replicates, defined as separate cultures or preparations initiated on different days. All reported data were consistent and reproducible across biological replicates, except for one outlier presented in SI Fig 4C, a condition for which 5 biological replicates were performed, and where the outlier was clearly disclosed.
Data exclusions	No data were excluded from our analyses.
Replication	Each central experiment was repeated at least three times, independently to generate biological replicates. For some parameterization experiments, technical replicates from experiments conducted on the same day were used. All attempts at replication were successful, except for one experiment reported in SI Fig. 4C, where 1 of 5 biological replicates deviated from the rest of our observations. This result is reported alongside the rest of the data.
Randomization	There were no additional covariates requiring controlled allocation, so all samples were treated identically except for the experimental condition, which was defined during sample preparation. Thus, sample allocation was not relevant to our study.
Blinding	Blinding of group allocation during data collection and analysis was not feasible because experimental conditions, such as cell density or initial nutrient concentration, were defined at the time of sample preparation. However, during data analysis, semi-automated analysis pipelines applied consistent thresholds and fixed parameters to data across experimental groups to minimize any subjective bias.

Reporting for specific materials, systems and methods

We require information from authors about some types of materials, experimental systems and methods used in many studies. Here, indicate whether each material, system or method listed is relevant to your study. If you are not sure if a list item applies to your research, read the appropriate section before selecting a response.

Materials & experimental systems

n/a	Involved in the study	
[x]	[ ]	Antibodies
[x]	[ ]	Eukaryotic cell lines
[x]	[ ]	Palaeontology and archaeology
[x]	[ ]	Animals and other organisms
[x]	[ ]	Clinical data
[x]	[ ]	Dual use research of concern
[x]	[ ]	Plants

Methods

n/a	Involved in the study	
[x]	[ ]	ChIP-seq
[x]	[ ]	Flow cytometry
[x]	[ ]	MRI-based neuroimaging

Plants

Seed stocks	N/A
Novel plant genotypes	N/A
Authentication	N/A
